# Intestinal immune responses to commensal and pathogenic protozoa

**DOI:** 10.3389/fimmu.2022.963723

**Published:** 2022-09-16

**Authors:** Aline Sardinha-Silva, Eliza V. C. Alves-Ferreira, Michael E. Grigg

**Affiliations:** Molecular Parasitology Section, Laboratory of Parasitic Diseases, National Institute of Allergy and Infectious Diseases, National Institutes of Health, Bethesda, MD, United States

**Keywords:** intestinal immunity, commensal, protozoa, blastocystis, *Cryptosporidium*, *Giardia*, *Toxoplasma*

## Abstract

The physical barrier of the intestine and associated mucosal immunity maintains a delicate homeostatic balance between the host and the external environment by regulating immune responses to commensals, as well as functioning as the first line of defense against pathogenic microorganisms. Understanding the orchestration and characteristics of the intestinal mucosal immune response during commensal or pathological conditions may provide novel insights into the mechanisms underlying microbe-induced immunological tolerance, protection, and/or pathogenesis. Over the last decade, our knowledge about the interface between the host intestinal mucosa and the gut microbiome has been dominated by studies focused on bacterial communities, helminth parasites, and intestinal viruses. In contrast, specifically how commensal and pathogenic protozoa regulate intestinal immunity is less well studied. In this review, we provide an overview of mucosal immune responses induced by intestinal protozoa, with a major focus on the role of different cell types and immune mediators triggered by commensal (*Blastocystis* spp. and *Tritrichomonas* spp.) and pathogenic (*Toxoplasma gondii*, *Giardia intestinalis*, *Cryptosporidium parvum*) protozoa. We will discuss how these various protozoa modulate innate and adaptive immune responses induced in experimental models of infection that benefit or harm the host.

## Introduction

Mucosal tissue is a physical barrier composed of biochemical and immunological components at the interface between the host and the external environment. Mucosal immunity plays a fundamental role in promoting tolerogenic immune responses in order to maintain homeostasis in addition to providing the first line of defense against pathogenic and non-pathogenic microorganisms (1).

**Figure 1:**
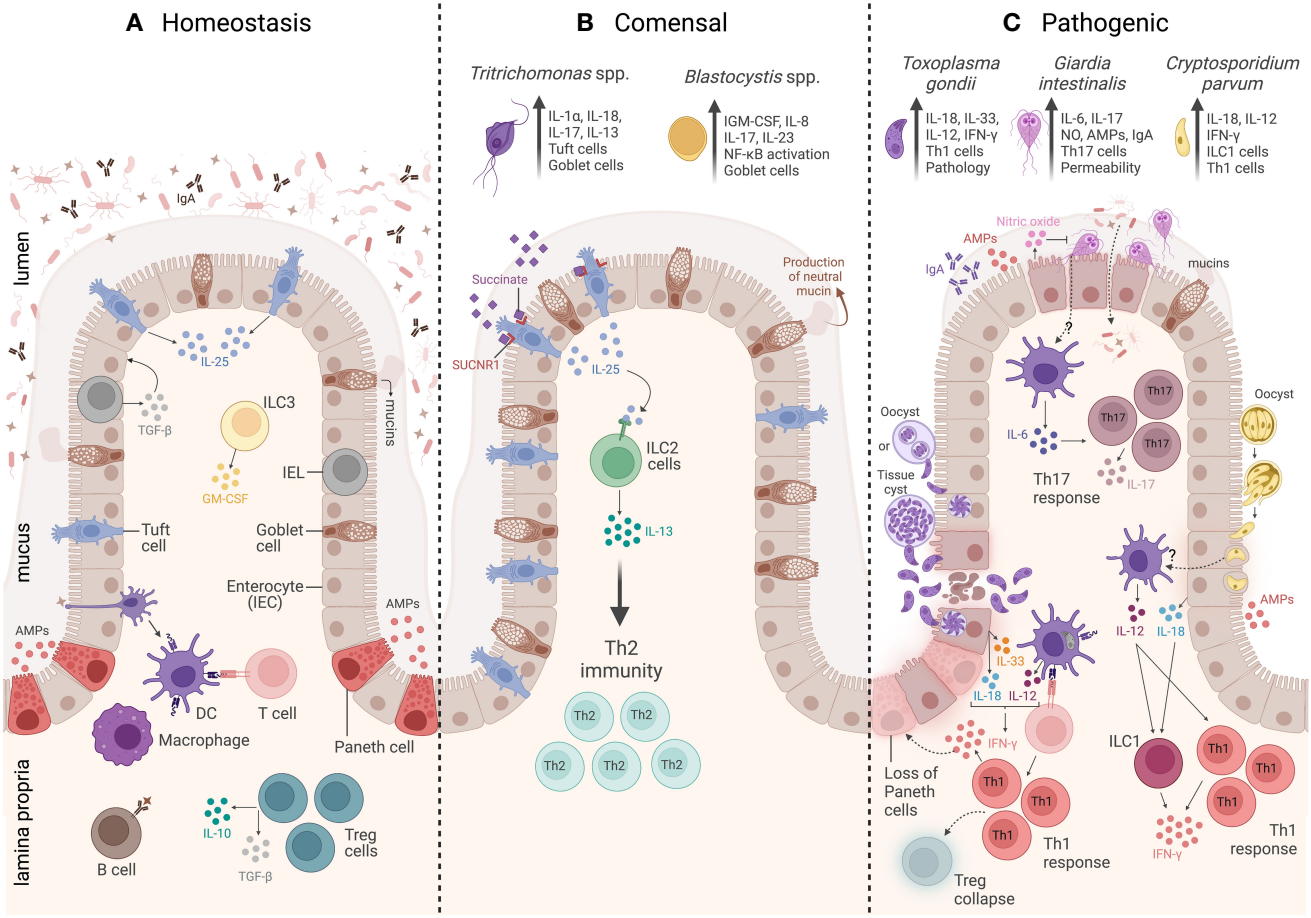
Overview of the intestinal mucosal immunity during homeostasis, commensal protozoa colonization, and pathogenic protozoa infection. **(A)** The homeostasis of a healthy intestine is maintained by several biochemical and cellular components that form a physical barrier, including a mucus layer that isolates the lumen from the epithelium and underlying the mucus. There is a group of specialized epithelial cells composed of enterocytes, goblet cells, and Paneth cells that secrete antimicrobial peptides (AMPs), tuft cells that constitutively express IL-25, and intraepithelial lymphocytes (IELs) that regulate epithelial growth through the secretion of TGF-β1. Underneath the epithelium is the lamina propria (LP), and the main cells involved in homeostasis are macrophages and dendritic cells (DCs) responsible for tolerance and T-cell activation. Innate lymphoid cell 3 (ILC3) is the main source of granulocyte–macrophage colony-stimulating factor (GM-CSF), while T regulatory cells (Tregs) are key to maintaining intestinal balance and tolerance through the secretion of IL-10 and TGF-β. **(B)** The commensal protozoa colonization is represented by two protists, *Tritrichomonas* ssp. and *Blastocystis* spp. In the small intestine, *Tritrichomonas* spp. secrete succinate molecules that bind in tuft cells SUCNR1 receptors, inducing these cells to release IL-25 resulting in ILC2 activation and IL-13 secretion, leading to Th2 response and goblet cells hyperplasia. Meanwhile, in the large intestine, *Tritrichomonas* spp. induce Th1 and Th17 immune responses by eliciting IL-1β, IL-18, and IL-17 cytokine release. *Blastocystis* colonization induces goblet cell hyperplasia, which leads to neutral mucin production and stimulates Th1/Th17 immune response, with IL-17 and IL-23 cytokine signatures. **(C)** The intestinal mucosal immunity to pathogenic protozoa infection is represented by three parasites: *Toxoplasma gondii*, *Giardia intestinalis*, and *Cryptosporidium parvum*. *T. gondii* infection is characterized by a strong Th1 immunity, tissue damage, and immunopathology. The immune response to this protozoan is characterized by high levels of IL-33- and IL-18-producing epithelial cells, as well as IL-12 production by DCs. All these three cytokines act together to induce CD4^+^ T cells producing IFN-γ. The infection is also characterized by Treg collapse and a loss of Paneth cells due to the high levels of IFN-γ. *G. intestinalis* infection is characterized by a Th17 immunity, elicited by IL-6-producing DCs and IL-17-producing CD4^+^ T cells. In addition, *G. intestinalis* increases intestinal epithelial permeability and microbial translocation, as well as enhances IgA, AMPs, nitric oxide (NO) levels, and mucin secretion. *C. parvum* infection is characterized by a Th1 immune response, which is associated with secretion of AMPs and IL-18 by epithelial cells, IL-12 by DCs, and IFN-γ production by both innate lymphoid cells 1 (ILC1) and CD4^+^ T cells. Created with BioRender.com.

The gastrointestinal tract is the largest mucosal tissue in the human body, which harbors a diverse community of commensals including prokaryotic bacteria, eukaryotic fungi, and protozoa, as well as other organisms such as intestinal viruses, helminth parasites, and pathogenic protozoa (**Figure 1**). Although commensals are commonly referred to as symbiotic microorganisms that are either non-pathogenic or beneficial (referred to as mutualists) to their host (2), it is increasingly evident that their presence plays an important role in reshaping the host immune system (3). Beyond their role in digestion and nutrient acquisition, the presence of commensal organisms is fundamental to maintaining intestinal homeostasis and modulating the development and maturation of the immune system, which is pivotal to effectively protecting the host against pathogenic organisms (4). Thus, intestinal mucosal immunity continuously functions to maintain a delicate balance to the commensal organisms, to avoid unnecessary inflammation to exogenous antigens (including food allergens) or damage-associated self-antigens, and, very importantly, to prevent the invasion or dissemination of pathogenic organisms (5–9). Remarkably, the lamina propria layer of the small intestine retains the highest concentration of immune cells, which mediate host protection against infective agents and also promote bystander inflammation and pathology (1).

Anatomically, the intestinal mucosa is composed of an epithelium, which consists of a folded single layer of epithelial cells, connected by tight junctions, that is superimposed by a mucus layer (10). It is organized into crypts (invaginations into the underlying mesenchyme) and villi (projections into the lumen) (11). The cellular composition of the epithelium includes enterocytes, goblet cells, Paneth cells, M cells, tuft cells, enteroendocrine cells, and intraepithelial lymphocytes (IELs) (12–14). Underlying the epithelium is the lamina propria (LP), formed by structural elements like fibroblasts, fibrocytes, vascular endothelia cells, muscle cells, and immune cells like macrophages, mast cells, dendritic cells (DCs), plasma cells, and B and T cells, which are responsible for maintaining homeostasis, immune tolerance to commensals, and a defensive barrier against invasive pathogenic agents (1, 13). The epithelium thus represents both a physical barrier and an important site for the production of cytokines, chemokines, and antimicrobial peptides that protect the host against various infections and danger signals (**Figure 1A**).

Epithelial cells are comprised of a diverse array of specialized cell types, which include goblet cells (professional mucin-producing cells responsible for forming a dense mucus gel layer) that function to protect and maintain healthy epithelial function; tuft cells are immune sentinels and taste-chemosensory cells that constitutively express IL-25; Paneth cells are important sources of antimicrobial peptides (AMPs; defensins and lysozyme) and growth factors, which are associated with host protection against enteric infections, maintenance of crypt stem cell activity, and intestinal homeostasis by their ability to regulate the microbiome in the lumen (11, 15–19). Finally, the IELs, composed of γδ T cells and CD8^+^ T cells (expressing the CD8α homodimer), are interspersed throughout the intestinal epithelial layer and regulate epithelial growth and homeostasis by their secretion of TGF-β1 (20). IELs do not require priming to respond since they are antigen-experienced T cells that release cytokines and kill target cells upon encountering specific antigens (21, 22).

In the lamina propria, macrophages, DCs, innate lymphoid cells (ILCs), natural killer (NK) cells, and T and B lymphocytes are the immune cells that work together to maintain intestinal mucosal homeostasis and barrier integrity. The myeloid compartment, represented by macrophages and DCs, is crucial for the maintenance of intestinal tolerance and the activation of T-cell immunity (23, 24). The recently described innate immune cell family of ILCs within the intestinal mucosa is comprised of three different groups, namely, ILC1, ILC2, and ILC3. Of note, ILC3s are critically involved in intestinal homeostasis and consist of the primary source of granulocyte–macrophage colony-stimulating factor (GM-CSF) (25–27). Intestinal microbes, by their ability to promote B-cell receptor (BCR) editing and the immunoglobulin diversification of resident B cells, also play an important role in the induction of tolerance against commensal antigens in the mucosa (28, 29). The intestinal mucosa is likewise home to the largest number of T cells in the body, comprised of a heterogeneous array of CD4^+^ and CD8^+^ T cells (29, 30). Most of these T cells in the mucosa possess an activated/memory phenotype, and their activation culminates in a rapid proliferative response (31). Among the most enriched T-cell populations in the gut are regulatory T cells (Tregs), which promote the maintenance of immune intestinal balance and tolerance, mainly by the secretion of IL-10 and TGF-β (32).

Understanding the orchestration and characteristics of the mucosal immune response during colonization by pathogenic or commensal organisms within the intestine may provide novel insights into the mechanisms underlying microbe-induced protection and/or immunopathology. Of note, most of our knowledge about the interactions between the host mucosal response and the gut microbiome, including the eukaryome, is dominated by studies focused on bacterial communities, helminth parasites, and intestinal viruses. Importantly, the impact of commensal and pathogenic eukaryotic protists that colonize the intestinal mucosa is less studied. In this review, we provide an overview of the current knowledge of protozoan-induced intestinal mucosal immunity, specifically, how commensal and pathogenic protozoa trigger the development and modulation of innate and adaptive immune responses in experimental models of infection and what the pathological consequences are for the host, if any.

## Mucosal immunity to commensal intestinal protozoa

Investigating the host gastrointestinal immune response to the microbiome is an emerging field. Most studies are focused on host responses generated against enteric bacterial communities. However, recent advances in amplicon-based next-generation sequencing (NGS) and metagenomics analysis have allowed a more comprehensive identification and description of the diverse micro-eukaryotes living within the mammalian microbiota, such as the fungi and protozoa that comprise the eukaryome (33). Large and small intestine-colonizing eukaryotes are commonly associated with pathogenic organisms such as *Toxoplasma gondii*, *Giardia* spp., and *Cryptosporidium* spp., which are discussed further in the second part of this review. However, enteric non-pathogenic commensal protozoa have also been identified by their ability to alter the gut–bacteriome diversity and to prime the host-immune response (34). Currently, relatively few investigations have examined gastrointestinal tract (GIT) immunity generated by the presence of protozoa that exist as enteric commensals. In this review, we specifically focus on *Blastocystis* spp. and *Tritrichomonas* spp. (**Figure 1B**).

### Blastocystis spp.

As reviewed by Parija and Jeremiah, *Blastocystis* spp. were first described by Brittan and Swayne during the 1849 cholera epidemic in London. To this day, the taxonomic classification of this organism remains controversial in the scientific community (35). In the early 1900s, Alexeieff and Emile Brumpt classified *Blastocystis* as a harmless saprophytic yeast of the intestinal tract. However, in 1967, Zierdt *et al.* reclassified the organism as a protist based on its morphology and phenotypic properties (36). Thus, after various observations over the decades, *Blastocystis* is currently classified as an anaerobic, non-invasive protist that colonizes the mammalian intestinal tract and belongs to the Stramenopiles, a clade of protists that group with algae, diatoms, and water molds (37). It possesses a high genetic diversity and a vast host range, including livestock and humans (38). Epidemiological estimates suggest that more than 1 billion people worldwide are colonized with *Blastocystis* spp., varying at 0.5%–23.1% in developed countries versus 22.1%–100% in resource-limited countries (39–41). *Blastocystis* spp. is currently comprised of 28 subtypes (STs), 12 of which are reported to colonize the GIT of humans (ST1–ST8, ST10, ST12, ST14, and ST16), but among them, only ST1–ST4 account for more than 90% of the human infections reported globally, whereas ST5–ST8, ST10, ST12, ST14, and ST16 are rarely found in human stool (42–46). *Blastocystis* has been detected in the human enteric microbiota in both healthy and disease conditions by different methods such as real-time PCR, amplicon-based NGS, and metagenomics. Its presence within the gut microbiota is associated with a shift in bacterial composition (47). The association between *Blastocystis* and the shift in microbial diversity and composition, as well as its effects on the host response remain to be determined. Although reports of this parasite are becoming more common in human gut studies, the direct *Blastocystis* mucosal immune response in the murine model is still under-explored.

Whether *Blastocystis* is pathogenic under some circumstances, for example, during host immune suppression, remains unclear, largely because screening for the presence of other known microorganisms such as viruses, protozoa, or bacteria has not been systematically performed (48, 49). Some studies have described a positive correlation between *Blastocystis* colonization and conditions such as irritable bowel syndrome or inflammatory bowel disease in humans (50, 51), whereas the high prevalence of this protist observed in healthy human guts has fueled a separate debate on whether this parasite is non-pathogenic and a commensal of the human GIT. Indeed, a sample cohort of healthy humans in Ireland identified that 56% of individuals were positive for *Blastocystis* by 18S rRNA PCR (52).

*In vitro* models suggest that *Blastocystis* can regulate host immune responses. Colonic epithelial cells exposed to *Blastocystis* Nand II strain released IL-8 and GM-CSF cytokines (53), possibly triggered by *Blastocystis* cysteine proteases, which are released by the zoonotic isolate WR1 (ST4) and activate the NF-κB transcription factor (54). In addition, *Blastocystis* ST7 (B isolate) can evade the immune response by downregulating inducible nitric oxide synthase (iNOS) (55) and degrading human secretory immunoglobulin A (IgA) (56).

Iguchi *et al.* determined that *Blastocystis* RN94-9 strain (a subtype 4 isolate from a laboratory rat) is not inconspicuous. Although rats experimentally infected with ST4 RN94-9 cysts largely failed to display pathogenic markers during colonization, such as weight loss, diarrhea symptoms, mucosal epithelium lesions, or inflammatory cell infiltrates (57), they did, however, induce type 1 proinflammatory cytokines such as IFN-γ, IL-12, and TNF-α and cause a mild goblet cell hyperplasia that was associated with an increase in the production of neutral mucins (57). Goblet cells are essential cells for maintaining gastrointestinal homeostasis. They synthesize neutral and acidic mucins to produce and sustain the enteric mucus layer, an innate barrier against pathogens (58). Alterations in the neutral and acidic mucin ratio have also been detected in rats infected by the parasitic nematode *Nippostrongylus brasiliensis* (59). While this imbalance in the production of neutral versus acidic mucins is thought to change mucus layer charge, whether it influences parasite-mucosa attachment is less clear.

In addition to the activation of a Th1 cytokine signature, *Blastocystis* modulates the *in vivo* Th17 cytokine immune response. Th17 immunity is strongly correlated with the clearance of extracellular parasites, inflammation, and auto-immune diseases (60). IL-17, IL-23, and IL-22 cytokines have a prominent role in Th17 subset activation (61). BALB/C mice infected with *Blastocystis* spp. developed significant increases in their IL-17 and IL-23 levels in the intestinal mucosa (62). In summary, *Blastocystis* appears to modulate the enteric host immune response by inducing specific Th1/Th17 cytokine production. Hence, colonization by *Blastocystis* may generate a level of bystander inflammation that could conceivably protect mice from pathogenic infections or make them more susceptible to certain auto-immune conditions. Therefore, studies to understand the kinetics, level of, and persistence of cytokine induction during *Blastocystis* colonization are needed. In addition, the data thus far investigating *Blastocystis* colonization *in vivo* certainly argue for the necessity to screen for the presence of commensal parasites in the gut in experimental workflows in order to gain a better understanding of their role in gastrointestinal disease models.

### Tritrichomonas spp.

*Tritrichomona*s spp. comprise a complex group of neglected gut-dwelling protists that have similar morphology but possess extant diversity. Studies within this group of parasites are limited, largely because of a lack of high-quality reference genomes or methods for axenic culture. Closely related species such as *Tritrichomona*s *muris*, *Tritrichomonas musculus*, and *Tritrichomonas rainier* have been discovered recently as common commensal protists that establish persistent, long-lived infections that impact the microbiome and mucosal immune homeostasis within the intestinal tract of wild and laboratory mice (63). Colonization of mice with these protists shows little to no evidence of epithelium lesion, blood cell infiltration, or other detectable gut-related disease symptoms, e.g., diarrhea, weight loss, or lethargy. Colonized mice do experience, however, a mild goblet cell hyperplasia and display significant changes in their mucosal immunity, which protect them from GIT bacterial infections but increase their susceptibility to inflammatory bowel-like diseases (64, 65). These studies certainly highlight the importance of screening the microbiota of laboratory mice for *Tritrichomonas* commensal infection.

In the healthy gut under homeostatic conditions, immune sentinels and taste-chemosensory cells called tuft cells are commonly found in low numbers within the epithelia of the small and large intestine in mice. However, in response to various parasitic infections, tuft cells are known to expand exponentially. For example, enteric non-pathogenic commensal protists, including *Tritrichomonas* spp., and pathogens such as nematodes (e.g., *N. brasiliensis*, *Heligmosomoides polygyrus*, and *Trichinella spiralis*) and trematodes (e.g., *Echinostoma caproni*) cause tuft cell activation and expansion, triggering mainly type 2 host immunity involving ILC2s (66–68).

Howitt *et al.* first described markedly different intestinal tuft cell numbers between specific pathogen-free BIH mice bred “in-house” versus BIH mice purchased from the Jackson Laboratory (JAX). When cecal contents from BIH mice were fed to the JAX mice, intestinal tuft cells proliferated to a level identified in the bred “in-house” BIH mice, and the transmissible component responsible for the phenotypic change was a single-celled protist that they referred to as *T. muris* (65). Two other contemporaneous studies likewise concluded that this and a related gut-dwelling commensal protist referred to as *T. musculus* alter immune cell homeostasis within the gut microenvironment, conferring the protection of colonized mice from mucosal bacterial infections at the expense of increasing their susceptibility to colitis and colorectal tumors (60, 67).

The Howitt study showed that shortly after *T. muris* colonization, tuft cells release the cytokine IL-25 and promote ILC2 activation and the secretion of IL-13, resulting in goblet cell hyperplasia and skewing the immune potential of the gut toward a Th2 response (65). Two years later, Nadjsombati *et al.* showed that the related parabasalid *T. rainier* also induced tuft cell expansion, ILC2 activation, and goblet cell hyperplasia in the small intestine of laboratory mice, similar to *T. muris* (69). Their work established that succinate secreted by *T. rainier* is a pivotal ligand sensed by the succinate receptor (SUCNR1) expressed on tuft cells and that this was sufficient to initiate a Th2-biased immune response in the small intestine (69). Succinate is a metabolite produced by hydrogenosomes, a mitochondria-like organelle that synthesizes ATP in anaerobic protists, including *Tritrichomonas* spp (70). This remarkable finding revealed for the first time that a metabolite unique to a eukaryotic microbe was able to directly activate type 2 immune cell responses *via* a tuft cell-ILC2 sensing and activation circuit (19).

Paradoxically enteric *Tritrichomonas* spp. infection also activates Th1 and Th17 immune cells and their signature cytokines during mouse colonization, in addition to initiating Th2-biased immune responses in the small intestine. In the large intestine specifically, *T. muris* stimulates a Th1-biased pro-inflammatory response, increasing IL-12/IL-23p40 and IFN-γ proteins to levels that exacerbate colitis in Rag1^−/−^ mice post-adoptive T-cell transfer (71). Chudnovskiy *et al.* also demonstrated a CD45^+^ hematopoietic cell expansion and the secretion of high levels of IgA in the large intestine of mice colonized with *T. musculus* (*T.mu*), a *T. muris*-related species (64). Like *T. muris*, *T.mu* also induced mild goblet cell hyperplasia and an exacerbated inflammatory response in colitis and tumorigenesis models. Within the cecal epithelium, high levels of IL-18 were released, the result of activating an ASC-dependent inflammasome, and this was important for the induction of colonic Th1 and Th17 immune responses in *T.mu*-colonized mice (64). The altered inflammatory state driven by the IL-18 release within the colonic epithelium was sufficient to protect mice from *Salmonella typhimurium* enterocolitis and established that the presence of *T.mu* significantly increased anti-bacterial defenses within the enteric mucosa (64). Recent work has identified that *T.mu* activates both the NLRP1B and NLRP3 inflammasomes and also causes a dramatic increase in luminal extracellular ATP levels (72). *T.mu* colonization likewise triggered IL-13 by ILC2s in the mucosa, as previously reported by Howitt *et al.* using *T. muris* (65). It also altered host glucose homeostasis by increasing gluconeogenesis, as well as increasing free choline production in the gut of infected hosts. The parasite metabolite that triggered this pathway is unknown, but there is evidence for a succinate-independent process (73). Altogether, the data described here emphasize the importance of investigating commensal protists in the gut microbiota in order to devise new therapeutic interventions to control inflammatory, pathogenic, and/or metabolic diseases.

## Intestinal mucosal immunity to pathogenic protozoa

A healthy and functional intestinal mucosa is both plastic and dynamic, which is necessary to maintain immune homeostasis and protect hosts from invading pathogenic organisms. However, dysregulated immune responses that result in chronic diseases within the gut epithelia have negative consequences for the host, including intestinal damage and a failure to control microbial pathogens (3). The following section summarizes the current knowledge on the intestinal mucosal immune response to the pathogenic protozoan parasites *T. gondii*, *Giardia intestinalis*, and *Cryptosporidium parvum* and describes the most relevant epithelial and immune cell populations involved in such responses (**Figure 1C**). Although these are all human parasites and their associated diseases are extremely relevant within the human population globally, our focus in this review is to describe the major features of the mucosal immune response triggered by these intestinal protozoa in experimental animal models to better understand the biology of their parasitism and highlight the parasite and host factors that specifically influence their pathogenicity.

### Toxoplasma gondii

*T. gondii* is an intracellular protozoan parasite capable of infecting essentially any warm-blooded animal. It is highly prevalent worldwide, with about 1/3 of the human population chronically infected with *T. gondii* parasites (74). The parasite is a leading cause of infectious blindness and is considered a life-threatening disease among the immunocompromised. It is also capable of causing abortion or birth defects during congenital infection (75, 76). *Toxoplasma* transmission occurs by oral ingestion of infectious tissue cysts or oocysts; hence, intestinal mucosal immunity constitutes the first line of defense and is one of the most important barriers against *T. gondii* (13, 77, 78). In the small intestine, *T. gondii* differentiates into tachyzoites, the rapidly replicating form of the parasite, which ultimately disseminates infection beyond the intestinal mucosa, colonizing other tissues, such as skeletal muscle and the central nervous system, before it differentiates into the bradyzoite form, which establishes latent infection in the form of tissue cysts (13). When in the gut, *Toxoplasma* alters the mucosal homeostatic balance and induces a cascade of immunological events involving components of both innate and adaptive immunity, characterized by a polarized Th1 immune response that induces high levels of IFN-γ and a collapse of Tregs (13, 79). The heightened inflammatory state has been shown to cause a Crohn’s disease-like enteritis, the result of an IFN-γ-mediated acute ileitis that occurs in some inbred mice (77, 80–83). Type 1 immunity is normally associated with host protection and is necessary for *T. gondii* clearance. However, in some *Toxoplasma* murine infection models, a dysregulated Th1 response can occur, which causes irreversible pathological alterations and inflammatory responses that promote severe tissue damage and host death (82–84). Here, we describe the mucosal-associated immune cell types that are induced during *T. gondii* infection in the gut with reference to well-described parasite factors that regulate the development of the *T. gondii*-associated mucosal immune response.

Gut epithelial and lamina propria cells are key players in host defense against *Toxoplasma* infection in the intestine. The gut epithelium is an important physical barrier and functions as the first line of innate defense against the parasite. In response to oral *Toxoplasma* infection, the gut epithelium releases alarmins IL-18 and IL-33 (both IL-1 family members), which synergize with IL-12 to promote protective IFN-γ-mediated immune responses that regulate *T. gondii* infection within the ileum. The release of IL-18 is caspase-1/11, ASC, and inflammasome sensor NLRP3- and NLRP1b-dependent (85). IL-18 plays a pivotal role in limiting parasite replication, as mice deficient in IL-18 or IL-18R experience 20–100-fold higher parasite loads and die acutely, compared to infected wild-type mice with the same genetic background (85, 86). It has also been reported that *T. gondii* actively crosses the gut epithelial barrier by invading, replicating, and lysing epithelial cells (78, 87). This process ruptures the gut epithelium and releases the damage-associated molecular pattern (DAMP) cytokine IL-33, which also synergizes with IL-12 to promote ILC1 production of IFN-γ and CCR2-dependent recruitment of inflammatory monocytes required for resistance to *T. gondii* (88). *T. gondii* tachyzoites are also released into the lamina propria compartment. Such collateral damage facilitates the translocation of bacteria across the gut epithelium, which exacerbates the inflammatory response and causes serious gut pathology (78, 89–92).

The presence of IFN-γ driven by *T. gondii* infection is also responsible for the rapid loss of Paneth cells within the host mucosa and results in a myriad of pleiotropic effects. Paneth cells are epithelial cells that secrete a suite of antimicrobial proteins and peptides in order to sustain mucosal homeostasis (15). In the presence of *Toxoplasma*, alterations in both membrane and mitochondrial integrity occur in Paneth, cells which activate an mTORC1-dependent cell death pathway (93–95). The loss of Paneth cells is also partially associated with the activation of TLR11 signaling in the gut (93). Likewise, IELs, another epithelial cell type comprised mainly of CD8α T cells, play a critical role in the initiation of tissue damage and immunopathology in response to *Toxoplasma* infection. The progression of pathological inflammation and necrosis is associated with the expansion of CCR2^hi^CD103^+^ IELs in the epithelium, which are principally induced by the presence of the CCL2 chemokine. Interestingly, CCR2^−/−^ mice experience diminished intestinal inflammation but had higher mortality rates, due largely to a failure to regulate *Toxoplasma* growth (96). Hence, IELs perform a critical function. Failure to regulate them precisely represents a double-edged sword: IELs secrete anti-inflammatory mediators, like TGF-β, to regulate mucosal inflammation in the GIT but can also expand to produce high levels of IFN-γ, which drives the immunopathology in response to *Toxoplasma* infection (77).

After crossing the epithelial barrier, parasites reach the LP, where immune cells are abundantly present. In the LP, innate immune cells, including inflammatory monocytes, dendritic cells, and NK cells, are critical players that both initiate and regulate the immune response against *T. gondii* infection (76). The myeloid compartment, specifically macrophages and dendritic cells, is responsible for sensing parasite antigens (pathogen-associated molecular patterns (PAMPs)) and rapidly respond by secreting IL-12, one of the major cytokines released in response to *Toxoplasma* infection. IL-12 principally drives the marked IFN-γ production by NK and T cells (97, 98). Among innate immune cells of non-myeloid origin, the role of ILCs during *T. gondii* infection is not well defined, so it remains unclear precisely how they contribute to the induction or regulation of mucosal immunity against *Toxoplasma*. Although it is known that ILC1 cells can secrete IFN-γ, the contribution of this cell type to the total aggregated IFN-γ levels during *Toxoplasma* infection is likely less relevant than that of CD4^+^ T cells. Indeed, López-Yglesias *et al.* demonstrated that in the absence of ILC1, Tbx21^−/−^ mice were still able to generate a robust IFN-γ response driven by CD4^+^ T cells (94). Of note, Wagage *et al.* suggested that RORyt^+^ ILC3 cells limit T-cell responses and pathology during *T. gondii* infection; however, they showed that ILC3 frequency is significantly decreased in the presence of the parasite (99). More studies on ILCs are required to fully understand their role in oral *T. gondii* infection models. Likewise, the role of neutrophils, another innate immune cell of myeloid origin, has not been systematically studied, so its role in contributing to mucosal immunity during *Toxoplasma* infection is not clear. Neutrophils rapidly migrate into the lamina propria during acute infection and can be detected within 3 days post-oral inoculation of *T. gondii* cysts (100, 101). Coombes *et al.* reported that tachyzoites preferentially infect infiltrating neutrophils, which function as reservoirs for *T. gondii* dissemination to other host tissues (102). Further studies are necessary to define the role of neutrophils during *Toxoplasma* intestinal infection.

One of the most important and well-studied cell types responding to *T. gondii* infection is DCs. These cells are the main source of IL-12 production and are central for the activation of T cells during both intraperitoneal and oral *T. gondii* infection. Several studies previously demonstrated that *T. gondii* proteins can directly activate Toll-like receptors (TLRs) to induce IL-12 production by DCs and macrophages. These include Micronemes 1 and 4 (TLR2/TLR4 agonists) and Profilin (TLR11/TLR12 agonists) (103–107). Although most of these studies were carried out using intraperitoneal infection models, it is known that profilin and TLR11 interactions also activate the intestinal innate immune response during oral infection with *T. gondii*. Benson *et al.* showed that mice deficient in TRL11 expression are more susceptible to *Toxoplasma* infection, the result of a marked reduction in IL-12 secretion and a partial defect in the generation of Th1 cells (108). In addition, recent studies have reported that IL-12—released during *Toxoplasma* infection—arises mainly from uninfected lamina propria DCs (CD11b^−^CD103^+^) rather than infected ones (109–111). It has been suggested that these cells respond to soluble parasite factors secreted prior to parasite invasion or parasite proteins that have been actively injected into DCs by *T. gondii* (109–111). Other parasite factors that regulate mucosal immunity include the Type II dense granule protein 15 (GRA15_II_), which activates NF-κB within innate immune infected cells and results in elevated IL-12 production, lower parasite burden, and less intestinal inflammation. This event was reported to occur only when the Type I rhoptry kinase 16 (ROP16_I_), another parasite factor that is involved in STAT3, STAT5, and STAT6 activation, was heterologously expressed within *T. gondii* Type II strains (112, 113). Hence, the combined expression of GRA15_II_ and ROP16_I_ in a Type II background strain conferred host resistance to acute oral infection by *T. gondii*. Finally, GRA24 is also released into the host cell cytoplasm to induce IL-12 production by triggering the activation of host mitogen-activated protein kinase p38 (p38 MAPK), which subsequently results in the transcription of the IL-12 gene (114–117). Additionally, different studies reported that there is a large CCR2-dependent influx of inflammatory monocytes into the lamina propria compartment during *Toxoplasma* infection, and IFN-γ-activated inflammatory monocytes are associated with the control of parasite replication and host resistance (111, 118). These cells are capable of producing IL-12 in the gut during infection, and this helps in the recruitment of protective Th1 cells in a CXCR3-dependent manner (119). Moreover, it is also reported that inflammatory monocytes are critical for *T. gondii* clearance by IFN-γ-dependent activation of iNOS/NOS2, the release of NO, and the activation of IRGs (immunity-related GTPases), which destroy the parasitophorous vacuole membrane (PVM) to limit parasite replication (92, 120, 121).

As mentioned before, *T. gondii* infection induces a very strong and polarized Th1 immune response, associated with a high frequency of IFN-γ-producing CD4^+^ T cells in murine models. Although Th1 immunity is required to control parasite replication, by inducing such a strong inflammatory response, in some mouse genetic backgrounds, it also drives a severe, and often lethal, intestinal immunopathology (81, 93, 122). López-Yglesias *et al.* demonstrated that even in the absence of T-bet, the transcriptional factor signature for Th1 cells, the IFN-γ-producing CD4^+^ T-cell response, is still markedly increased in Tbx21^−/−^ mice, suggesting that the immunopathogenesis during infection can be driven by a T-bet-independent pathway (94). Additionally, the *T. gondii*-Th1-induced tissue damage in the intestinal mucosa is associated with the collapse of resident Tregs and a subsequent reduction of IL-10 levels during infection (79). This phenomenon is associated with the conversion of Tregs into Tbet^+^ IFN-γ-producing cells and favors the development of a Th1-driven Crohn’s disease-like immunopathology (79, 123). Finally, Rachinel *et al.* suggested that the acute ileitis driven by *T. gondii* is associated with the expression of the parasite immunodominant antigen SAG1 (surface antigen 1), which elicits a CD4^+^ T cell-dependent lethal inflammatory process in the oral B6 mouse model (124). Although Th1 responses dominate during *T. gondii* infection, a few studies suggest that IL-17A- and IL-22-secreting Th17 cells also play an important role in maintaining persistent levels of antimicrobial peptide production, given that mice deficient in class I-restricted T cell-associated molecule (CRTAM) expression on T cells failed to control *T. gondii*-driven immunopathology and microbial translocation (125, 126).

Among all the intestinal pathogenic protozoa parasites that will be discussed in this review, it is notable that the area of study regarding *T. gondii* biology has evolved dramatically during the past few years. In addition, advances in gene editing tools and experimental models are continually contributing to our understanding of important host–parasite interactions that impact host immune responses against *T. gondii*. However, the role of many parasite factors and their interaction impacting the development of host intestinal immunity remains unknown. Ultimately, further studies are required to comprehensively elucidate the underpinnings of the protective immune response that can be elicited to revert immunopathology and confer a host-protective effect by reducing or preventing *T. gondii*-driven acute ileitis.

### Giardia intestinalis

The intestinal extracellular protozoan parasite *G. intestinalis* (syn. *Giardia duodenalis* and *Giardia lamblia*) is highly prevalent worldwide and can infect all mammals, including humans, domestic animals, and wildlife. Giardiasis is considered one of the most prevalent enteric diseases caused by a protozoan parasite, with estimates ranging from 2%–5% to 20%–30% of *Giardia*-infected people in high and low-middle-income countries, respectively. Approximately 280 million new cases are documented every year worldwide (127–129). Immunocompetent individuals typically resolve the infection spontaneously within a few weeks after exposure, and some people never develop any symptoms (130, 131). However, susceptible individuals often exhibit a myriad of different gastrointestinal symptoms, including abdominal cramps, nausea, and diarrhea (132). These clinical symptoms are frequently associated with the development of malabsorption syndrome and with children’s development and growth impairment, especially in chronic and recurrent *Giardia* infections (133–135). Recent data suggest that giardiasis is one of the four main contributors to stunting in children (135–137). Transmission of *Giardia* occurs through the oral–fecal route with the ingestion of parasite cysts present in contaminated water or food (138), highlighting the importance of the intestinal mucosal immune response triggered by *Giardia* infection, which will be discussed here.

Within the gastrointestinal environment, *Giardia* cysts release trophozoites, the parasite form that adheres to the epithelial monolayer to colonize the small intestine, especially the duodenum. Trophozoites attach to the microvilli in the epithelium to replicate and complete their life cycle, but this alters mucosal homeostasis and induces several host responses. In mice, *Giardia* infection is mainly characterized by a protective Th17 immune response, with high levels of IgA secretion, and the presence of IL-17 is associated with parasite clearance (139, 140).

The intestinal epithelium functions as a physical barrier to protect from invading pathogens reaching the deeper layers of the mucosa. Although *Giardia* trophozoites do not invade the mucosa or submucosa layers in immunocompetent organisms, many studies suggest that *Giardia* trophozoites attach to the intestinal epithelial cells (IECs) and rupture the epithelial barrier by disrupting tight junction proteins within the IECs, such as claudins and occludin, which increases intestinal permeability, facilitating the entry and spread of enteric pathogens (141–143). In addition to functioning as a physical barrier to avoid parasite invasion in the lamina propria, the intestinal epithelial layer is responsible for producing various chemicals, such as nitric oxide (NO), and antimicrobial peptides that inhibit *Giardia* replication in the epithelium (144). However, *Giardia*-produced arginine deiminase (ADI) is thought to reduce the availability of arginine in the gut, which negatively affects NO production by enterocytes (144). In addition, Stadelmann *et al.* reported that arginine consumption by *Giardia* is also associated with decreased proliferation of intestinal epithelial cells during infection (145). Epithelial Paneth cells produce and release α-defensins, an AMP that, in combination with NO from nitric oxide synthase (NOS2), acts to control *Giardia* burden and eliminate infection (146). *Giardia* infection also upregulates mucus production during intestinal infection (147–149).

*Giardia* infection results in the rupture of the epithelial barrier, and this event is associated with the release of chemokines that recruit immune cells required for the protective response against *Giardia*. The epithelial disruption also promotes microbial translocation and the release of parasite antigens within the lamina propria, which induces the activation of recruited and resident myeloid cells (140, 150–152). However, studies regarding the specific role of macrophages and dendritic cells during *Giardia* infection in mice are sparse in the literature. It is known that DCs are one of the main sources of IL-6, and in the absence of this cytokine, IL-6 knockout mice fail to control *Giardia* replication during *in vivo* infections (153–155). It is reported that *Giardia* can induce a cytokine profile that is less inflammatory, and it seems that the reduction of proinflammatory cytokines is mediated by the activation of TLR2 signaling in DCs and macrophages (156, 157). Paradoxically, infection in mice that lack TLR2 is associated with reduced parasite burden and less *Giardia*-associated pathology (156). Further studies are necessary to understand the specific role of myeloid cells during *Giardia in vivo* infection.

*Giardia* induces the recruitment of mast cells into the small intestine lamina propria, and at the site of infection, these cells degranulate and release histamines and protease-1 (MMCP-1) (158, 159). Li *et al.* described that MMCP-1 interacts with cholecystokinin (CCK), and this interaction results in more intestinal contractility, which is thought to be associated with cramps, one of the most common symptoms noted by infected people (128, 159). The literature suggests that mast cells are recruited *via* activation of the complement lectin pathway since mice lacking mannose-binding lectin 2 (MBL2) and complement factor 3a receptor (C3aR) expression are impaired in mast cell recruitment during *Giardia* infection (160). Additionally, the expression of MBL2 in the intestinal mucosa is dependent on *Giardia*-induced IL-17 (147).

As mentioned before, *Giardia* infection induces the differentiation and expansion of IL-17-producing CD4^+^ T cells, which confer a host-protective effect by limiting parasite replication. It is reported that the control of infection is dependent on T cells but independent of Th1 and Th2 immunity (139, 140, 161). However, as is the case for many other pathogens, a protective immune response that limits the *Giardia* burden may also generate significant pathology. Known sequelae associated with *Giardia* infection in mice are accelerated intestinal transit contributing to parasite-induced diarrhea, muscle hypercontractility, mast cell activation, and cramping (158, 159, 162, 163). Lastly, activated CD8^+^ T effector cells (CD44^hi^ and CD69^hi^) mediate tissue damage and microvillous shortening, which is responsible for disaccharidase enzyme (sucrase and lactase) deficiency and malabsorption of electrolytes, nutrients, and water (156, 164). The mechanism of CD8^+^ T-cell activation during *Giardia* infection, however, remains unclear but is thought to be influenced by the intestinal microbiota (156).

While there has been significant progress in understanding the host–parasite interaction and the immune response induced by *Giardia*, further studies are needed to determine which parasite factors specifically trigger innate immune activation, as well as the signals implicated in Th17 activation within the intestinal mucosa, and how CD8^+^ T cells become activated to induce pathology. Finally, new studies are required to better understand the nature of the protective immune response induced by *Giardia*, which is associated with a reduced risk of developing a severe diarrheal disease in children, and whether *Giardia* parasitism may be relevant or harmful in the context of a co-infection with another enteric pathogen.

### Cryptosporidium parvum

*Cryptosporidium* spp. are epicellular protozoan parasites that colonize the gastrointestinal tract of mammals. The species *C. parvum* and *Cryptosporidium hominis* cause the majority of human infections globally (165, 166). In immunocompetent individuals, cryptosporidiosis is usually a self-limiting infection, with mild (abdominal pain and diarrhea) or absent symptoms, but this parasite can cause serious diseases in immunodeficient patients leading to severe, life-threatening diarrhea (166, 167). The enteric disease is also the second leading cause of severe diarrheal illness in children under 5 years old, with an estimated 60,000 deaths per year worldwide (168). In addition, it is responsible for affecting the growth and development of children. The limited availability of therapeutic treatments and the total lack of a vaccine represent a challenge for disease prevention (168). Parasite transmission occurs through the oral–fecal route upon the ingestion of infectious oocysts (169). Among the three pathogenic protozoa discussed in this review, *Cryptosporidium* infection and its associated mucosal immunity are much less explored by the scientific community, mainly because of a lack of experimental tools, including the ability to readily cultivate the parasite life cycle in the laboratory and the limited availability of animal models. Nevertheless, recent advances adapting *Cryptosporidium* to mice are facilitating new insight and permitting investigation of the intestinal mucosal immune response during *in vivo* infection.

Within the intestinal lumen, released sporozoites adhere to the epithelial plasmalemma to form a parasitophorous vacuole, a compartment isolated from both the lumen and host cell cytoplasm where parasites replicate and promote colonization of the small intestine. Cryptosporidiosis is characterized by crypt hyperplasia, villous atrophy, and diffuse shortening or loss of brush border microvilli, which results in malabsorption, dehydration, and diarrhea (166, 167). *Cryptosporidium* initiates an inflammatory process by activating epithelial cells (IECs) and inducing the secretion of “alarmin” cytokines (IL-8, TNF-α, and IL-1β) and chemokines (CCL2, CCL5, CXCL1, CXCL8, CXCL9, CXCL10, etc.) (170, 171). This event triggers the recruitment of effector immune cells to the site of infection and inhibits parasite adhesion to the mucosal epithelium (169–172). Various other experimental studies have concluded that production of β-defensin antimicrobial peptides, as well as cytokines, chemokines, and prostaglandin E2 by IECs, occurs *via* activation of the TLR2/TLR4/NF-κB signaling pathway during *Cryptosporidium* infection (171, 173, 174). Moreover, NF-κB activation induces the expression of anti-apoptotic factors that prolongs the life of IECs, promoting parasite replication and the propagation of the infection (175, 176).

The secretion of chemokines by *Cryptosporidium*-activated IECs is responsible for the recruitment of inflammatory monocytes, macrophages, and NK cells to the site of infection, and the presence of local IL-18 and IL-12 induces a synergistic activation of macrophages and NK cells to secrete high levels of IFN-γ in infected neonatal mice (170, 177). Depletion of NK cells was associated with an exacerbated infection in neonatal mice (178). Furthermore, Choudhry *et al.* reported that the neutralization of IL-18 during *Cryptosporidium* infection, after *in vivo* treatment with anti-IL-18 antibodies, resulted in decreased IFN-γ expression in Rag2^−/−^γc^−/−^ mice (177). Different studies using transgenic mice have shown that deficiencies in either Th1 cytokines, such as IL-12, IL-18, and IFN-γ, or inflammasome components (caspase-1) are associated with a more susceptible or sometimes lethal phenotype to *Cryptosporidium* infection (179–183). Additionally, a recent study has demonstrated that *Cryptosporidium* is recognized by the inflammasome sensor NOD-like receptor family pyrin domain containing 6 (NLRP6), which activates enterocytes to release bioactive IL-18 and is required for parasite control (183). Finally, the synergistic secretion of IL-18 and IL-12 by activated enterocytes stimulates ILCs to produce IFN-γ, and this event promotes parasite control by enterocytes (184). Thus, the combination of IL-12, IL-18, and IFN-γ as well as inflammasome activation seem to be associated with a protective mechanism against infection.

Given the fact that IFN-γ suppresses *Cryptosporidium* infection and controls parasite replication, it has been described that CD4^+^ T cells are also essential for parasite elimination and the establishment of an effective immune response following infection (166). Many studies have reported that neutralization of IFN-γ in Rag2^−/−^ or SCID mice was associated with increased *Cryptosporidium* burden, persistent diarrhea, and progress of intestinal pathology, sometimes leading to mouse death (179, 185–189). Intriguingly, McDonald *et al.* showed evidence that during early *Cryptosporidium* infection, mice secreted intestinal IL-4. They showed that susceptibility to infection was increased and associated with higher production of oocysts when mice were treated with anti-IL-4 antibodies. Their data suggest that early IL-4 can promote a protective, Th1-mediated mucosal immune response that inhibits parasite development (190). The concomitant neutralization of IL-4 and IL-5 was also shown to increase oocyst shedding in infected mice (191). Lastly, the role of CD8^+^ T cells and B cells during *Cryptosporidium* infection is less clear. Although it has been reported that they expand during infection, whether they are essential for the clearance of infection remains enigmatic (192, 193). Additionally, while parasite-specific IgM and IgG levels have been shown to increase during infection, they do not prevent the host from secondary infection, although specific antibodies are known to reduce oocyst shedding in a reinfection event (194, 195).

Despite the difficulties of conducting experimental studies in the *Cryptosporidium* field, knowledge is quickly evolving given the development of new technologies and lab strategies to improve parasite culture, life cycle, and experimental models. These new techniques are allowing previously unanswered questions about the biology of *Cryptosporidium* parasitism to be explored, including how parasite proteins can be delivered directly to the cytosol of infected host cells (196). Still, many questions remain unanswered, such as what parasite factors are recognized by the host cells that initiate the innate mucosal immune response or what extracellular or intracellular receptors are involved in parasite recognition.

## Microbial dysbiosis: A common feature between commensal and pathogenic protozoa

The identification of the gut eukaryome, including some intestinal protozoa discussed in this review, has allowed scientists not only to characterize their interaction with the mammalian host and their capacity to re-shape intestinal immunity but also to investigate their association with a large group of bacterial communities that inhabit the mammalian gut and specifically how these interactions might alter host gut homeostasis and promote disease pathology (33, 64).

Apart from the ability to sense intestinal immunity, all enteric protozoa discussed in this review share a mutual capacity to alter the host gut microbiome. These microeukaryotes, independent of their pathogenic or commensal status, alter the diversity of the microbiome during colonization of the intestine, which can alter mucosal immune homeostasis and promote diseases (34). For instance, recent studies suggest that colonization with *Tritrichomonas* is associated with decreased microbial diversity (197), whereas colonization with *Blastocystis* increases microbial diversity and is associated with the expansion of Clostridia and depletion of Enterobacteriaceae (198), which reduces inflammation by promoting a more healthy gut microbiota. However, some enteric protozoa, such as *Toxoplasma* (91, 92, 199), *Giardia* (200–202), and *Cryptosporidium* (203, 204), promote a shift in microbial community structure associated with dysbiosis, which exacerbates diseases and facilitates bacterial translocation (205). For example, *T. gondii* causes a profound expansion of Enterobacteriaceae (especially *Escherichia coli*), resulting in dysbiosis and increased immune-driven pathology in C57BL/6J mice (92). Additionally, Wang *et al.* showed that host-derived nitrate generated by the activation of the immune response against *T. gondii* promoted the overgrowth of Enterobacteriaceae. This occurs due to the activation of the IFN-γ/STAT1/iNOS pathway in macrophages, which is the same pathway associated with parasite control during infection (92). Indeed, it has been shown that the impaired activation of macrophages, by the absence of STAT1 signaling or deficiency in CCR2 expression or by blocking TNF-α with neutralizing antibodies, can induce a marked reduction of Enterobacteriaceae expansion (90). Interestingly, the influence of enteric protozoa on the gut microbiome may also be regulated by host genetic background, because BALB/cJ mice infected with *T. gondii* do not develop enteric dysbiosis, nor do they promote nitrate-dependent overgrowth of Enterobacteriaceae, and they do not develop a dysregulated mucosal immune response and lethal ileitis, as seen in C57BL/6J mice (206–208).

Nevertheless, the common ability of intestinal commensal and pathogenic protozoa to induce microbial shifts during colonization results in different microbiomes and disease profiles, which can help to explain the divergence in the type of mucosal immune response that develops and whether a protozoan parasite is considered a commensal or a pathogen. Finally, the type of microbial shift that occurs and the mucosal immune response that develops not only help to explain how variations in disease can occur but may also determine whether the parasite is cleared, establishes a persistent infection, and/or is transmissible.

## What makes a protist commensal or pathogenic?

The distinction between a host-associated commensal (defined as a microbe eating at the same table) and a pathogen (a microbe that causes harm) is often obscure. Depending on the context, some commensals can cause diseases (i.e., *Blastocystis*), whereas some pathogens can persist without causing overt diseases (i.e., *Cryptosporidium* and *Giardia*). The late Stanley Falkow perhaps said it best, that some microbes, he termed “commensal pathogens”, can persist as natural members of the indigenous flora but possess an innate ability to cross anatomical barriers, invade tissues, or breach host defenses that ordinarily limit the survival or replication of other microbes or commensals (209). Perhaps the central question is rather how to limit these invasive properties in order to reduce disease potential. As mentioned above, many factors are likely to influence the relative pathogenicity of intestinal microeukaryotes, including 1) microbial shifts that expand or decrease specific classes of bacteria (taxa) that activate or regulate the immune response, 2) the host species and its genetic background, 3) the presence of microbe-associated molecular patterns (MAMPs) that exist as virulence factors that induce inflammation and/or pathology, or 4) the induction of chronic inflammation or granulomatous reactions to contain the infection. The microbial shift induced by pathogenic protozoa often differs from that induced by commensal protozoa. The molecular basis for this is varied; for example, the diversity of host or bacterial-associated metabolites can skew the immune response to either a regulatory profile or a more inflammatory one (210–213). Moreover, the bioproducts from enteric protozoa, such MAMPs, especially present in pathogenic microbes, can activate pathological aspects of the host immune response, and the inability to regulate this response may cause diseases (3). For instance, many bacteria can be both commensal and pathogenic, such as *E. coli*, a gram-negative versatile bacterium that is a commensal organism of the healthy gut microbiome but, through the acquisition of virulence genes by horizontal gene transfer (HGT) and/or accumulation of mutations in the genome, can convert from a commensal bacterium to a pathogenic one capable of causing a wide range of extraintestinal diseases (214–219). Similar mechanisms are known to occur in other organisms, where many species or strains initially not pathogenic evolved as pathogens due to the acquisition of virulence genes or the expansion of multi-gene families and/or epigenetic regulation mechanisms that promote invasive properties (220, 221). In addition, pathogenicity could also be associated with the relative abundance of specific microbes in the gastrointestinal tract, where their uncontrolled growth promotes the transition of microeukaryotes into pathogenic ones no longer controlled by host immunity (222). Thus, many protists can be considered both commensal and pathogenic; what determines their relative pathogenicity is a combination of different factors that together combine to determine their virulence potential.

## Future perspectives

Throughout this review, it became clear and fascinating that studies focused on the investigation of intestinal protozoan biology have progressed extensively over the last decade. The discovery of new commensal protozoa and how they can reshape the host intestinal immune response has been extremely important in understanding the alteration of gut–bacteriome diversity and how this can affect the priming of mucosal immunity in general, which ultimately impacts (or defines) how the host will respond to gastrointestinal insults, such as parasitic infections or inflammatory diseases. Nevertheless, further studies are required to characterize these mechanisms in more detail and shed important insight specifically on how intestinal protozoa reshape intestinal immune potential in naïve hosts. Along with the discoveries about commensal protozoa, the advances in gene editing tools and experimental models are continually contributing to our understanding of the host–parasite interaction that impacts host mucosal immune responses against pathogenic protists. However, the precise parasite factors and how they trigger host intestinal immunity remain understudied. This review has discussed our current understanding of the intestinal mucosal immune homeostatic landscape and how it shifts in response to colonization by commensal or pathogenic protozoa. We believe that future work in this area will continue to shed important perspectives on the mechanisms underlying microbe-induced protection and/or immunopathology and will identify new biomarkers for therapeutic intervention to clear parasites and control disease.

## Author contributions

Conceptualization: AS-S. Funding acquisition: MG. Writing – original draft: AS-S and EA-F. Writing – review and editing: AS-S and MG. All authors contributed to the article, read, and approved the submitted version.

## Funding

This study was supported by the Division of Intramural Research (DIR), National Institute of Allergy and Infectious Diseases (NIAID), NIH.

## Conflict of interest

The authors declare that the research was conducted in the absence of any commercial or financial relationships that could be construed as a potential conflict of interest.

The handling editor declared a shared affiliation with the authors at the time of review.

## Publisher’s note

All claims expressed in this article are solely those of the authors and do not necessarily represent those of their affiliated organizations, or those of the publisher, the editors and the reviewers. Any product that may be evaluated in this article, or claim that may be made by its manufacturer, is not guaranteed or endorsed by the publisher.

## References

[1] McGhee JR Fujihashi K. Inside the mucosal immune system. PloS Biol (2012) 10(9):e1001397. doi: 10.1371/journal.pbio.1001397 23049482 PMC3457930

[2] Martens EC Neumann M Desai MS. Interactions of commensal and pathogenic microorganisms with the intestinal mucosal barrier. Nat Rev Microbiol (2018) 16(8):457–70. doi: 10.1038/s41579-018-0036-x 29904082

[3] Kasper LH Buzoni-Gatel D. Ups and downs of mucosal cellular immunity against protozoan parasites. Infect Immun (2001) 69(1):1–8. doi: 10.1128/IAI.69.1.1-8.2001 11119482 PMC97848

[4] Kabat AM Srinivasan N Maloy KJ. Modulation of immune development and function by intestinal microbiota. Trends Immunol (2014) 35(11):507–17. doi: 10.1016/j.it.2014.07.010 PMC6485503 25172617

[5] Kayama H Takeda K. Regulation of intestinal homeostasis by innate and adaptive immunity. Int Immunol (2012) 24(11):673–80. doi: 10.1093/intimm/dxs094 22962437

[6] Perez-Lopez A Behnsen J Nuccio SP Raffatellu M. Mucosal immunity to pathogenic intestinal bacteria. Nat Rev Immunol (2016) 16(3):135–48. doi: 10.1038/nri.2015.17 26898110

[7] Okumura R Takeda K. Maintenance of intestinal homeostasis by mucosal barriers. Inflammation Regen. (2018) 38:5. doi: 10.1186/s41232-018-0063-z PMC5879757 29619131

[8] Tokuhara D Kurashima Y Kamioka M Nakayama T Ernst P Kiyono H. A comprehensive understanding of the gut mucosal immune system in allergic inflammation. Allergol Int (2019) 68(1):17–25. doi: 10.1016/j.alit.2018.09.004 30366757

[9] Rodriguez-Sillke Y Visekruna A Glauben R Siegmund B Steinhoff U. Recognition of food antigens by the mucosal and systemic immune system: Consequences for intestinal development and homeostasis. Int J Med Microbiol (2021) 311(3):151493. doi: 10.1016/j.ijmm.2021.151493 33652373

[10] Grondin JA Kwon YH Far PM Haq S Khan WI. Mucins in intestinal mucosal defense and inflammation: Learning from clinical and experimental studies. Front Immunol (2020) 11:2054. doi: 10.3389/fimmu.2020.02054 33013869 PMC7500085

[11] Gerbe F Legraverend C Jay P. The intestinal epithelium tuft cells: specification and function. Cell Mol Life Sci (2012) 69(17):2907–17. doi: 10.1007/s00018-012-0984-7 PMC3417095 22527717

[12] van der Flier LG Clevers H. Stem cells, self-renewal, and differentiation in the intestinal epithelium. Annu Rev Physiol (2009) 71:241–60. doi: 10.1146/annurev.physiol.010908.163145 18808327

[13] Cohen SB Denkers EY. Border maneuvers: deployment of mucosal immune defenses against toxoplasma gondii. Mucosal Immunol (2014) 7(4):744–52. doi: 10.1038/mi.2014.25 24717355

[14] Allaire JM Crowley SM Law HT Chang SY Ko HJ Vallance BA. The intestinal epithelium: Central coordinator of mucosal immunity. Trends Immunol (2018) 39(9):677–96. doi: 10.1016/j.it.2018.04.002 29716793

[15] Bevins CL Salzman NH. Paneth cells, antimicrobial peptides and maintenance of intestinal homeostasis. Nat Rev Microbiol (2011) 9(5):356–68. doi: 10.1038/nrmicro2546 21423246

[16] Sato T van Es JH Snippert HJ Stange DE Vries RG van den Born M. Paneth cells constitute the niche for Lgr5 stem cells in intestinal crypts. Nature. (2011) 469(7330):415–8. doi: 10.1038/nature09637 PMC3547360 21113151

[17] Adolph TE Tomczak MF Niederreiter L Ko HJ Bock J Martinez-Naves E. Paneth cells as a site of origin for intestinal inflammation. Nature. (2013) 503(7475):272–6. doi: 10.1038/nature12599 PMC3862182 24089213

[18] Clevers HC Bevins CL. Paneth cells: maestros of the small intestinal crypts. Annu Rev Physiol (2013) 75:289–311. doi: 10.1146/annurev-physiol-030212-183744 23398152

[19] Ting HA von Moltke J. The immune function of tuft cells at gut mucosal surfaces and beyond. J Immunol (2019) 202(5):1321–9. doi: 10.4049/jimmunol.1801069 PMC6383804 30782851

[20] Cheroutre H Lambolez F Mucida D. The light and dark sides of intestinal intraepithelial lymphocytes. Nat Rev Immunol (2011) 11(7):445–56. doi: 10.1038/nri3007 PMC3140792 21681197

[21] Mayassi T Jabri B. Human intraepithelial lymphocytes. Mucosal Immunol (2018) 11(5):1281–9. doi: 10.1038/s41385-018-0016-5 PMC6178824 29674648

[22] Sim GK. Intraepithelial lymphocytes and the immune system. Adv Immunol (1995) 58:297–343. doi: 10.1016/S0065-2776(08)60622-7 7741030

[23] Stagg AJ. Intestinal dendritic cells in health and gut inflammation. Front Immunol (2018) 9:2883. doi: 10.3389/fimmu.2018.02883 30574151 PMC6291504

[24] Sun T Nguyen A Gommerman JL. Dendritic cell subsets in intestinal immunity and inflammation. J Immunol (2020) 204(5):1075–83. doi: 10.4049/jimmunol.1900710 32071090

[25] Spits H Cupedo T. Innate lymphoid cells: emerging insights in development, lineage relationships, and function. Annu Rev Immunol (2012) 30:647–75. doi: 10.1146/annurev-immunol-020711-075053 22224763

[26] Mortha A Chudnovskiy A Hashimoto D Bogunovic M Spencer SP Belkaid Y. Microbiota-dependent crosstalk between macrophages and ILC3 promotes intestinal homeostasis. Science. (2014) 343(6178):1249288. doi: 10.1126/science.1249288 24625929 PMC4291125

[27] Bennett MS Round JL Leung DT. Innate-like lymphocytes in intestinal infections. Curr Opin Infect Dis (2015) 28(5):457–63. doi: 10.1097/QCO.0000000000000189 PMC4925623 26270655

[28] Wesemann DR Portuguese AJ Meyers RM Gallagher MP Cluff-Jones K Magee JM. Microbial colonization influences early b-lineage development in the gut lamina propria. Nature. (2013) 501(7465):112–5. doi: 10.1038/nature12496 PMC3807868 23965619

[29] Kabat AM Pott J Maloy KJ. The mucosal immune system and its regulation by autophagy. Front Immunol (2016) 7:240. doi: 10.3389/fimmu.2016.00240 27446072 PMC4916208

[30] Mowat AM Agace WW. Regional specialization within the intestinal immune system. Nat Rev Immunol (2014) 14(10):667–85. doi: 10.1038/nri3738 25234148

[31] Shale M Schiering C Powrie F. CD4(+) T-cell subsets in intestinal inflammation. Immunol Rev (2013) 252(1):164–82. doi: 10.1111/imr.12039 PMC3736165 23405904

[32] Harrison OJ Powrie FM. Regulatory T cells and immune tolerance in the intestine. Cold Spring Harb Perspect Biol (2013) 5(7):a018341. doi: 10.1101/cshperspect.a018341 23818502 PMC3685893

[33] Lukes J Stensvold CR Jirku-Pomajbikova K Wegener Parfrey L. Are human intestinal eukaryotes beneficial or commensals? PloS Pathog (2015) 11(8):e1005039. doi: 10.1371/journal.ppat.1005039 26270819 PMC4536199

[34] del Campo J Bass D Keeling PJ. The eukaryome: Diversity and role of microeukaryotic organisms associated with animal hosts. Funct Ecol (2020) 34(10):2045–54. doi: 10.1111/1365-2435.13490

[35] Parija SC Jeremiah S. Blastocystis: Taxonomy, biology and virulence. Trop Parasitol (2013) 3(1):17–25. doi: 10.4103/2229-5070.113894 23961437 PMC3745665

[36] Zierdt CH Rude WS Bull BS. Protozoan characteristics of blastocystis hominis. Am J Clin Pathol (1967) 48(5):495–501. doi: 10.1093/ajcp/48.5.495 6058384

[37] Silberman JD Sogin ML Leipe DD Clark CG. Human parasite finds taxonomic home. Nature. (1996) 380(6573):398. doi: 10.1038/380398a0 8602239

[38] Alfellani MA Taner-Mulla D Jacob AS Imeede CA Yoshikawa H Stensvold CR. Genetic diversity of blastocystis in livestock and zoo animals. Protist. (2013) 164(4):497–509. doi: 10.1016/j.protis.2013.05.003 23770574

[39] Nithyamathi K Chandramathi S Kumar S. Predominance of blastocystis sp. infection among school children in peninsular Malaysia. PloS One (2016) 11(2):e0136709. doi: 10.1371/journal.pone.0136709 26914483 PMC4767405

[40] Osman M El Safadi D Cian A Benamrouz S Nourrisson C Poirier P. Prevalence and risk factors for intestinal protozoan infections with cryptosporidium, giardia, blastocystis and dientamoeba among schoolchildren in Tripoli, Lebanon. PloS Negl Trop Dis (2016) 10(3):e0004496. doi: 10.1371/journal.pntd.0004496 26974335 PMC4790957

[41] Ning CQ Hu ZH Chen JH Ai L Tian LG. Epidemiology of blastocystis infection from 1990 to 2019 in China. Infect Dis Poverty. (2020) 9(1):168. doi: 10.1186/s40249-020-00779-z 33380335 PMC7772921

[42] Alfellani MA Stensvold CR Vidal-Lapiedra A Onuoha ES Fagbenro-Beyioku AF Clark CG. Variable geographic distribution of blastocystis subtypes and its potential implications. Acta Trop (2013) 126(1):11–8. doi: 10.1016/j.actatropica.2012.12.011 23290980

[43] Khademvatan S Masjedizadeh R Yousefi-Razin E Mahbodfar H Rahim F Yousefi E. PCR-based molecular characterization of blastocystis hominis subtypes in southwest of Iran. J Infect Public Health (2018) 11(1):43–7. doi: 10.1016/j.jiph.2017.03.009 28404232

[44] Stensvold CR Clark CG. Pre-empting pandora's box: Blastocystis subtypes revisited. Trends Parasitol (2020) 36(3):229–32. doi: 10.1016/j.pt.2019.12.009 32001133

[45] Higuera A Herrera G Jimenez P Garcia-Corredor D Pulido-Medellin M Bulla-Castaneda DM. Identification of multiple blastocystis subtypes in domestic animals from Colombia using amplicon-based next generation sequencing. Front Vet Sci (2021) 8:732129. doi: 10.3389/fvets.2021.732129 34504891 PMC8421793

[46] Maloney JG Jang Y Molokin A George NS Santin M. Wide genetic diversity of blastocystis in white-tailed deer (Odocoileus virginianus) from Maryland, USA. Microorganisms. (2021) 9(6):1343. doi: 10.3390/microorganisms9061343 34205799 PMC8233720

[47] Deng L Wojciech L Gascoigne NRJ Peng G Tan KSW. New insights into the interactions between blastocystis, the gut microbiota, and host immunity. PloS Pathog (2021) 17(2):e1009253. doi: 10.1371/journal.ppat.1009253 33630979 PMC7906322

[48] Stenzel DJ Boreham PF. Blastocystis hominis revisited. Clin Microbiol Rev (1996) 9(4):563–84. doi: 10.1128/CMR.9.4.563 PMC172910 8894352

[49] Chabe M Lokmer A Segurel L. Gut Protozoa: Friends or foes of the human gut microbiota? Trends Parasitol (2017) 33(12):925–34. doi: 10.1016/j.pt.2017.08.005 28870496

[50] Shirvani G Fasihi-Harandi M Raiesi O Bazargan N Zahedi MJ Sharifi I. Prevalence and molecular subtyping of blastocystis from patients with irritable bowel syndrome, inflammatory bowel disease and chronic urticaria in Iran. Acta Parasitol (2020) 65(1):90–6. doi: 10.2478/s11686-019-00131-y 31602552

[51] Pena S Carrasco G Rojas P Castillo D Ozaki LS Mercado R. Determination of subtypes of blastocystis sp. in Chilean patients with and without inflammatory bowel syndrome, a preliminary report. Parasite Epidemiol Control. (2020) 8:e00125. doi: 10.1016/j.parepi.2019.e00125 31890923 PMC6926359

[52] Scanlan PD Stensvold CR Rajilic-Stojanovic M Heilig HG De Vos WM O'Toole PW. The microbial eukaryote blastocystis is a prevalent and diverse member of the healthy human gut microbiota. FEMS Microbiol Ecol (2014) 90(1):326–30. doi: 10.1111/1574-6941.12396 25077936

[53] Long HY Handschack A Konig W Ambrosch A. Blastocystis hominis modulates immune responses and cytokine release in colonic epithelial cells. Parasitol Res (2001) 87(12):1029–30. doi: 10.1007/s004360100494 11763434

[54] Puthia MK Lu J Tan KS. Blastocystis ratti contains cysteine proteases that mediate interleukin-8 response from human intestinal epithelial cells in an NF-kappaB-dependent manner. Eukaryot Cell (2008) 7(3):435–43. doi: 10.1128/EC.00371-07 PMC2268520 18156286

[55] Mirza H Wu Z Kidwai F Tan KS. A metronidazole-resistant isolate of blastocystis spp. is susceptible to nitric oxide and downregulates intestinal epithelial inducible nitric oxide synthase by a novel parasite survival mechanism. Infect Immun (2011) 79(12):5019–26. doi: 10.1128/IAI.05632-11 PMC3232666 21930763

[56] Puthia MK Vaithilingam A Lu J Tan KS. Degradation of human secretory immunoglobulin a by blastocystis. Parasitol Res (2005) 97(5):386–9. doi: 10.1007/s00436-005-1461-0 16151742

[57] Iguchi A Yoshikawa H Yamada M Kimata I Arizono N. Expression of interferon gamma and proinflammatory cytokines in the cecal mucosa of rats experimentally infected with blastocystis sp. strain RN94-9. Parasitol Res (2009) 105(1):135–40. doi: 10.1007/s00436-009-1373-5 19255785

[58] Kim JJ Khan WI. Goblet cells and mucins: role in innate defense in enteric infections. Pathogens. (2013) 2(1):55–70. doi: 10.3390/pathogens2010055 25436881 PMC4235714

[59] Koninkx JF Mirck MH Hendriks HG Mouwen JM van Dijk JE. Nippostrongylus brasiliensis: histochemical changes in the composition of mucins in goblet cells during infection in rats. Exp Parasitol (1988) 65(1):84–90. doi: 10.1016/0014-4894(88)90109-9 3338549

[60] Oukka M. Th17 cells in immunity and autoimmunity. Ann Rheum Dis (2008) 67 Suppl 3:iii26–9. doi: 10.1136/ard.2008.098004 19022809

[61] Stockinger B Veldhoen M. Differentiation and function of Th17 T cells. Curr Opin Immunol (2007) 19(3):281–6. doi: 10.1016/j.coi.2007.04.005 17433650

[62] Wu LY Fu RJ Lu ZC Tang LL Zhang F Liu DY. [Expressions and significance of IL-17 and IL-23 in intestinal mucosa of mice infected with blastocystis hominis]. Zhongguo Xue Xi Chong Bing Fang Zhi Za Zhi. (2012) 24(6):676–80. 23593840

[63] Guillen N. Eukaryome: Emerging field with profound translational potential. Cham: Springer (2020).

[64] Chudnovskiy A Mortha A Kana V Kennard A Ramirez JD Rahman A. Host-protozoan interactions protect from mucosal infections through activation of the inflammasome. Cell. (2016) 167(2):444–56.e14. doi: 10.1016/j.cell.2016.08.076 27716507 PMC5129837

[65] Howitt MR Lavoie S Michaud M Blum AM Tran SV Weinstock JV. Tuft cells, taste-chemosensory cells, orchestrate parasite type 2 immunity in the gut. Science. (2016) 351(6279):1329–33. doi: 10.1126/science.aaf1648 PMC5528851 26847546

[66] von Moltke J Ji M Liang HE Locksley RM. Tuft-cell-derived IL-25 regulates an intestinal ILC2-epithelial response circuit. Nature. (2016) 529(7585):221–5. doi: 10.1038/nature16161 PMC4830391 26675736

[67] Gerbe F Jay P. Intestinal tuft cells: epithelial sentinels linking luminal cues to the immune system. Mucosal Immunol (2016) 9(6):1353–9. doi: 10.1038/mi.2016.68 27554294

[68] Rajeev S Sosnowski O Li S Allain T Buret AG McKay DM. Enteric tuft cells in host-parasite interactions. Pathogens. (2021) 10(9):1163. doi: 10.3390/pathogens10091163 34578195 PMC8467374

[69] Nadjsombati MS McGinty JW Lyons-Cohen MR Jaffe JB DiPeso L Schneider C. Detection of succinate by intestinal tuft cells triggers a type 2 innate immune circuit. Immunity. (2018) 49(1):33–41 e7. doi: 10.1016/j.immuni.2018.06.016 30021144 PMC6084797

[70] Muller M Mentel M van Hellemond JJ Henze K Woehle C Gould SB. Biochemistry and evolution of anaerobic energy metabolism in eukaryotes. Microbiol Mol Biol Rev (2012) 76(2):444–95. doi: 10.1128/MMBR.05024-11 PMC3372258 22688819

[71] Escalante NK Lemire P Cruz Tleugabulova M Prescott D Mortha A Streutker CJ. The common mouse protozoa tritrichomonas muris alters mucosal T cell homeostasis and colitis susceptibility. J Exp Med (2016) 213(13):2841–50. doi: 10.1084/jem.20161776 PMC5154950 27836928

[72] Chiaranunt P Burrows K Ngai L Cao EY Liang H Tai SL. NLRP1B and NLRP3 control the host response following colonization with the commensal protist tritrichomonas musculis. J Immunol (2022) 208(7):1782–9. doi: 10.4049/jimmunol.2100802 35256512

[73] Kou Y Meng L Zhang S Zheng X Liu M Xu S. A murine commensal protozoan influences host glucose homeostasis by facilitating free choline generation. Appl Environ Microbiol (2022) 88(6):e0241321. doi: 10.1128/aem.02413-21 35080909 PMC8939315

[74] Flegr J Prandota J Sovickova M Israili ZH. Toxoplasmosis–a global threat. correlation of latent toxoplasmosis with specific disease burden in a set of 88 countries. PloS One (2014) 9(3):e90203. doi: 10.1371/journal.pone.0090203 24662942 PMC3963851

[75] McLeod R Van Tubbergen C Montoya JG Petersen E. Human Toxoplasma infection. Toxoplasma gondii: The model apicomplexan, 2nd ed. Elsevier: Amsterdam, The Netherlands, (2014). pp. 99–159.

[76] Snyder LM Denkers EY. From initiators to effectors: Roadmap through the intestine during encounter of toxoplasma gondii with the mucosal immune system. Front Cell Infect Microbiol (2020) 10:614701. doi: 10.3389/fcimb.2020.614701 33505924 PMC7829212

[77] Kasper L Courret N Darche S Luangsay S Mennechet F Minns L. Toxoplasma gondii and mucosal immunity. Int J Parasitol (2004) 34(3):401–9. doi: 10.1016/j.ijpara.2003.11.023 15003499

[78] Schulthess J Fourreau D Darche S Meresse B Kasper L Cerf-Bensussan N. Mucosal immunity in toxoplasma gondii infection. Parasite. (2008) 15(3):389–95. doi: 10.1051/parasite/2008153389 18814712

[79] Oldenhove G Bouladoux N Wohlfert EA Hall JA Chou D Dos Santos L. Decrease of Foxp3+ treg cell number and acquisition of effector cell phenotype during lethal infection. Immunity. (2009) 31(5):772–86. doi: 10.1016/j.immuni.2009.10.001 PMC2814877 19896394

[80] Sher A Coffman RL. Regulation of immunity to parasites by T cells and T cell-derived cytokines. Annu Rev Immunol (1992) 10:385–409. doi: 10.1146/annurev.iy.10.040192.002125 1590992

[81] Liesenfeld O Kosek J Remington JS Suzuki Y. Association of CD4+ T cell-dependent, interferon-gamma-mediated necrosis of the small intestine with genetic susceptibility of mice to peroral infection with toxoplasma gondii. J Exp Med (1996) 184(2):597–607. doi: 10.1084/jem.184.2.597 8760813 PMC2192709

[82] Denkers EY Gazzinelli RT. Regulation and function of T-cell-mediated immunity during toxoplasma gondii infection. Clin Microbiol Rev (1998) 11(4):569–88. doi: 10.1128/CMR.11.4.569 PMC88897 9767056

[83] Dupont CD Christian DA Hunter CA. Immune response and immunopathology during toxoplasmosis. Semin Immunopathol (2012) 34(6):793–813. doi: 10.1007/s00281-012-0339-3 22955326 PMC3498595

[84] Egan CE Cohen SB Denkers EY. Insights into inflammatory bowel disease using toxoplasma gondii as an infectious trigger. Immunol Cell Biol (2012) 90(7):668–75. doi: 10.1038/icb.2011.93 PMC4094106 22064707

[85] Gorfu G Cirelli KM Melo MB Mayer-Barber K Crown D Koller BH. Dual role for inflammasome sensors NLRP1 and NLRP3 in murine resistance to toxoplasma gondii. mBio (2014) 5(1):e01117-13. doi: 10.1128/mBio.01117-13 24549849 PMC3944820

[86] Munoz M Eidenschenk C Ota N Wong K Lohmann U Kuhl AA. Interleukin-22 induces interleukin-18 expression from epithelial cells during intestinal infection. Immunity. (2015) 42(2):321–31. doi: 10.1016/j.immuni.2015.01.011 25680273

[87] Speer CA Dubey JP. Ultrastructure of early stages of infections in mice fed toxoplasma gondii oocysts. Parasitology. (1998) 116(Pt 1):35–42. doi: 10.1017/S0031182097001959 9481772

[88] Clark JT Christian DA Gullicksrud JA Perry JA Park J Jacquet M. IL-33 promotes innate lymphoid cell-dependent IFN-γ production required for innate immunity to Toxoplasma gondii. Elife (2021) 10 :e65614. doi: 10.7554/eLife.65614 33929319 PMC8121546

[89] Heimesaat MM Bereswill S Fischer A Fuchs D Struck D Niebergall J. Gram-negative bacteria aggravate murine small intestinal Th1-type immunopathology following oral infection with toxoplasma gondii. J Immunol (2006) 177(12):8785–95. doi: 10.4049/jimmunol.177.12.8785 17142781

[90] Craven M Egan CE Dowd SE McDonough SP Dogan B Denkers EY. Inflammation drives dysbiosis and bacterial invasion in murine models of ileal crohn's disease. PloS One (2012) 7(7):e41594. doi: 10.1371/journal.pone.0041594 22848538 PMC3404971

[91] Molloy MJ Grainger JR Bouladoux N Hand TW Koo LY Naik S. Intraluminal containment of commensal outgrowth in the gut during infection-induced dysbiosis. Cell Host Microbe (2013) 14(3):318–28. doi: 10.1016/j.chom.2013.08.003 PMC4806337 24034617

[92] Wang S El-Fahmawi A Christian DA Fang Q Radaelli E Chen L. Infection-induced intestinal dysbiosis is mediated by macrophage activation and nitrate production. mBio (2019) 10(3):e00935-19. doi: 10.1128/mBio.00935-19 31138751 PMC6538788

[93] Raetz M Hwang SH Wilhelm CL Kirkland D Benson A Sturge CR. Parasite-induced TH1 cells and intestinal dysbiosis cooperate in IFN-gamma-dependent elimination of paneth cells. Nat Immunol (2013) 14(2):136–42. doi: 10.1038/ni.2508 PMC3552073 23263554

[94] Lopez-Yglesias AH Burger E Araujo A Martin AT Yarovinsky F. T-Bet-independent Th1 response induces intestinal immunopathology during toxoplasma gondii infection. Mucosal Immunol (2018) 11(3):921–31. doi: 10.1038/mi.2017.102 PMC6179443 29297501

[95] Araujo A Safronova A Burger E Lopez-Yglesias A Giri S Camanzo ET. IFN-γ mediates paneth cell death via suppression of mTOR. Elife. (2021) 10 :e60478. doi: 10.7554/eLife.60478 34633285 PMC8570691

[96] Egan CE Craven MD Leng J Mack M Simpson KW Denkers EY. CCR2-dependent intraepithelial lymphocytes mediate inflammatory gut pathology during toxoplasma gondii infection. Mucosal Immunol (2009) 2(6):527–35. doi: 10.1038/mi.2009.105 PMC2860785 19741601

[97] Mahmoudzadeh S Nozad Charoudeh H Marques CS Bahadory S Ahmadpour E. The role of IL-12 in stimulating NK cells against toxoplasma gondii infection: a mini-review. Parasitol Res (2021) 120(7):2303–9. doi: 10.1007/s00436-021-07204-w 34110502

[98] Sasai M Pradipta A Yamamoto M. Host immune responses to toxoplasma gondii. Int Immunol (2018) 30(3):113–9. doi: 10.1093/intimm/dxy004 29408976

[99] Wagage S Harms Pritchard G Dawson L Buza EL Sonnenberg GF Hunter CA. The group 3 innate lymphoid cell defect in aryl hydrocarbon receptor deficient mice is associated with T cell hyperactivation during intestinal infection. PloS One (2015) 10(5):e0128335. doi: 10.1371/journal.pone.0128335 26010337 PMC4444139

[100] Sukhumavasi W Egan CE Warren AL Taylor GA Fox BA Bzik DJ. TLR adaptor MyD88 is essential for pathogen control during oral toxoplasma gondii infection but not adaptive immunity induced by a vaccine strain of the parasite. J Immunol (2008) 181(5):3464–73. doi: 10.4049/jimmunol.181.5.3464 PMC2614926 18714019

[101] Gregg B Taylor BC John B Tait-Wojno ED Girgis NM Miller N. Replication and distribution of toxoplasma gondii in the small intestine after oral infection with tissue cysts. Infect Immun (2013) 81(5):1635–43. doi: 10.1128/IAI.01126-12 PMC3647985 23460516

[102] Coombes JL Charsar BA Han SJ Halkias J Chan SW Koshy AA. Motile invaded neutrophils in the small intestine of toxoplasma gondii-infected mice reveal a potential mechanism for parasite spread. Proc Natl Acad Sci U S A. (2013) 110(21):E1913–22. doi: 10.1073/pnas.1220272110 PMC3666704 23650399

[103] Sardinha-Silva A Mendonca-Natividade FC Pinzan CF Lopes CD Costa DL Jacot D. The lectin-specific activity of toxoplasma gondii microneme proteins 1 and 4 binds toll-like receptor 2 and 4 n-glycans to regulate innate immune priming. PloS Pathog (2019) 15(6):e1007871. doi: 10.1371/journal.ppat.1007871 31226171 PMC6608980

[104] Yarovinsky F Zhang D Andersen JF Bannenberg GL Serhan CN Hayden MS. TLR11 activation of dendritic cells by a protozoan profilin-like protein. Science. (2005) 308(5728):1626–9. doi: 10.1126/science.1109893 15860593

[105] Kucera K Koblansky AA Saunders LP Frederick KB de la Cruz EM Ghosh S. Structure-based analysis of toxoplasma gondii profilin: a parasite-specific motif is required for recognition by toll-like receptor 11. J Mol Biol (2010) 403(4):616–29. doi: 10.1016/j.jmb.2010.09.022 PMC2957522 20851125

[106] Koblansky AA Jankovic D Oh H Hieny S Sungnak W Mathur R. Recognition of profilin by toll-like receptor 12 is critical for host resistance to toxoplasma gondii. Immunity. (2013) 38(1):119–30. doi: 10.1016/j.immuni.2012.09.016 PMC3601573 23246311

[107] Raetz M Kibardin A Sturge CR Pifer R Li H Burstein E. Cooperation of TLR12 and TLR11 in the IRF8-dependent IL-12 response to toxoplasma gondii profilin. J Immunol (2013) 191(9):4818–27. doi: 10.4049/jimmunol.1301301 PMC3805684 24078692

[108] Benson A Pifer R Behrendt CL Hooper LV Yarovinsky F. Gut commensal bacteria direct a protective immune response against toxoplasma gondii. Cell Host Microbe (2009) 6(2):187–96. doi: 10.1016/j.chom.2009.06.005 PMC2746820 19683684

[109] Koshy AA Dietrich HK Christian DA Melehani JH Shastri AJ Hunter CA. Toxoplasma co-opts host cells it does not invade. PloS Pathog (2012) 8(7):e1002825. doi: 10.1371/journal.ppat.1002825 22910631 PMC3406079

[110] Chen L Christian DA Kochanowsky JA Phan AT Clark JT Wang S. The toxoplasma gondii virulence factor ROP16 acts in cis and trans, and suppresses T cell responses. J Exp Med (2020) 217(3):e20181757. doi: 10.1084/jem.20181757 31961916 PMC7062521

[111] Cohen SB Denkers EY. Impact of toxoplasma gondii on dendritic cell subset function in the intestinal mucosa. J Immunol (2015) 195(6):2754–62. doi: 10.4049/jimmunol.1501137 PMC4561193 26283477

[112] Jensen KD Wang Y Wojno ED Shastri AJ Hu K Cornel L. Toxoplasma polymorphic effectors determine macrophage polarization and intestinal inflammation. Cell Host Microbe (2011) 9(6):472–83. doi: 10.1016/j.chom.2011.04.015 PMC3131154 21669396

[113] Jensen KD Hu K Whitmarsh RJ Hassan MA Julien L Lu D. Toxoplasma gondii rhoptry 16 kinase promotes host resistance to oral infection and intestinal inflammation only in the context of the dense granule protein GRA15. Infect Immun (2013) 81(6):2156–67. doi: 10.1128/IAI.01185-12 PMC3676013 23545295

[114] Kim L Del Rio L Butcher BA Mogensen TH Paludan SR Flavell RA. p38 MAPK autophosphorylation drives macrophage IL-12 production during intracellular infection. J Immunol (2005) 174(7):4178–84. doi: 10.4049/jimmunol.174.7.4178 15778378

[115] Braun L Brenier-Pinchart MP Yogavel M Curt-Varesano A Curt-Bertini RL Hussain T. A toxoplasma dense granule protein, GRA24, modulates the early immune response to infection by promoting a direct and sustained host p38 MAPK activation. J Exp Med (2013) 210(10):2071–86. doi: 10.1084/jem.20130103 PMC3782045 24043761

[116] Mercer HL Snyder LM Doherty CM Fox BA Bzik DJ Denkers EY. Toxoplasma gondii dense granule protein GRA24 drives MyD88-independent p38 MAPK activation, IL-12 production and induction of protective immunity. PloS Pathog (2020) 16(5):e1008572. doi: 10.1371/journal.ppat.1008572 32413093 PMC7255617

[117] Mukhopadhyay D Arranz-Solis D Saeij JPJ. Toxoplasma GRA15 and GRA24 are important activators of the host innate immune response in the absence of TLR11. PloS Pathog (2020) 16(5):e1008586. doi: 10.1371/journal.ppat.1008586 32453782 PMC7274473

[118] Dunay IR Damatta RA Fux B Presti R Greco S Colonna M. Gr1(+) inflammatory monocytes are required for mucosal resistance to the pathogen toxoplasma gondii. Immunity. (2008) 29(2):306–17. doi: 10.1016/j.immuni.2008.05.019 PMC2605393 18691912

[119] Cohen SB Maurer KJ Egan CE Oghumu S Satoskar AR Denkers EY. CXCR3-dependent CD4(+) T cells are required to activate inflammatory monocytes for defense against intestinal infection. PloS Pathog (2013) 9(10):e1003706. doi: 10.1371/journal.ppat.1003706 24130498 PMC3795032

[120] Khan IA Schwartzman JD Matsuura T Kasper LH. A dichotomous role for nitric oxide during acute toxoplasma gondii infection in mice. Proc Natl Acad Sci U S A. (1997) 94(25):13955–60. doi: 10.1073/pnas.94.25.13955 PMC28414 9391134

[121] Hunn JP Feng CG Sher A Howard JC. The immunity-related GTPases in mammals: a fast-evolving cell-autonomous resistance system against intracellular pathogens. Mamm Genome (2011) 22(1-2):43–54. doi: 10.1007/s00335-010-9293-3 21052678 PMC3438224

[122] Burger E Araujo A Lopez-Yglesias A Rajala MW Geng L Levine B. Loss of paneth cell autophagy causes acute susceptibility to toxoplasma gondii-mediated inflammation. Cell Host Microbe (2018) 23(2):177–90.e4. doi: 10.1016/j.chom.2018.01.001 29358083 PMC6179445

[123] Suzuki Y Sher A Yap G Park D Neyer LE Liesenfeld O. IL-10 is required for prevention of necrosis in the small intestine and mortality in both genetically resistant BALB/c and susceptible C57BL/6 mice following peroral infection with toxoplasma gondii. J Immunol (2000) 164(10):5375–82. doi: 10.4049/jimmunol.164.10.5375 10799901

[124] Rachinel N Buzoni-Gatel D Dutta C Mennechet FJ Luangsay S Minns LA. The induction of acute ileitis by a single microbial antigen of toxoplasma gondii. J Immunol (2004) 173(4):2725–35. doi: 10.4049/jimmunol.173.4.2725 15294991

[125] Cortez VS Cervantes-Barragan L Song C Gilfillan S McDonald KG Tussiwand R. CRTAM controls residency of gut CD4+CD8+ T cells in the steady state and maintenance of gut CD4+ Th17 during parasitic infection. J Exp Med (2014) 211(4):623–33. doi: 10.1084/jem.20130904 PMC3978276 24687959

[126] Cervantes-Barragan L Cortez VS Wang Q McDonald KG Chai JN Di Luccia B. CRTAM protects against intestinal dysbiosis during pathogenic parasitic infection by enabling Th17 maturation. Front Immunol (2019) 10:1423. doi: 10.3389/fimmu.2019.01423 31312200 PMC6614434

[127] Savioli L Smith H Thompson A. Giardia and cryptosporidium join the 'Neglected diseases initiative'. Trends Parasitol (2006) 22(5):203–8. doi: 10.1016/j.pt.2006.02.015 16545611

[128] Solaymani-Mohammadi S Singer SM. Giardia duodenalis: the double-edged sword of immune responses in giardiasis. Exp Parasitol (2010) 126(3):292–7. doi: 10.1016/j.exppara.2010.06.014 PMC2933943 20599999

[129] Yoder JS Gargano JW Wallace RM Beach MJ Centers for Disease C, Prevention. Giardiasis surveillance–united states, 2009-2010. MMWR Surveill Summ. (2012) 61(5):13–23. 22951494

[130] Garzon M Pereira-da-Silva L Seixas J Papoila AL Alves M Ferreira F. Association of enteric parasitic infections with intestinal inflammation and permeability in asymptomatic infants of sao tome island. Pathog Glob Health (2017) 111(3):116–27. doi: 10.1080/20477724.2017.1299831 PMC5445637 28279129

[131] Kraft MR Klotz C Bucker R Schulzke JD Aebischer T. Giardia's epithelial cell interaction *In vitro*: Mimicking asymptomatic infection? Front Cell Infect Microbiol (2017) 7:421. doi: 10.3389/fcimb.2017.00421 29018775 PMC5622925

[132] Solaymani-Mohammadi S. Mucosal defense against giardia at the intestinal epithelial cell interface. Front Immunol (2022) 13:817468. doi: 10.3389/fimmu.2022.817468 35250996 PMC8891505

[133] Mahmud MA Chappell C Hossain MM Habib M Dupont HL. Risk factors for development of first symptomatic giardia infection among infants of a birth cohort in rural Egypt. Am J Trop Med Hyg (1995) 53(1):84–8. doi: 10.4269/ajtmh.1995.53.84 7625540

[134] Al-Mekhlafi HM Al-Maktari MT Jani R Ahmed A Anuar TS Moktar N. Burden of giardia duodenalis infection and its adverse effects on growth of schoolchildren in rural Malaysia. PloS Negl Trop Dis (2013) 7(10):e2516. doi: 10.1371/journal.pntd.0002516 24205426 PMC3814875

[135] Rogawski ET Bartelt LA Platts-Mills JA Seidman JC Samie A Havt A. Determinants and impact of giardia infection in the first 2 years of life in the MAL-ED birth cohort. J Pediatr Infect Dis Soc (2017) 6(2):153–60. doi: 10.1093/jpids/piw082 PMC5907871 28204556

[136] Bartelt LA Sartor RB. Advances in understanding giardia: determinants and mechanisms of chronic sequelae. F1000Prime Rep (2015) 7:62. doi: 10.12703/P7-62 26097735 PMC4447054

[137] Rogawski ET Liu J Platts-Mills JA Kabir F Lertsethtakarn P Siguas M. Use of quantitative molecular diagnostic methods to investigate the effect of enteropathogen infections on linear growth in children in low-resource settings: longitudinal analysis of results from the MAL-ED cohort study. Lancet Glob Health (2018) 6(12):E1319–E28. doi: 10.1016/S2214-109X(18)30351-6 PMC6227248 30287125

[138] Adam RD. Biology of giardia lamblia. Clin Microbiol Rev (2001) 14(3):447–75. doi: 10.1128/CMR.14.3.447-475.2001 PMC88984 11432808

[139] Saghaug CS Sornes S Peirasmaki D Svard S Langeland N Hanevik K. Human memory CD4+ T cell immune responses against giardia lamblia. Clin Vaccine Immunol (2016) 23(1):11–8. doi: 10.1128/CVI.00419-15 PMC4711086 26376930

[140] Singer SM Fink MY Angelova VV. Recent insights into innate and adaptive immune responses to giardia. Adv Parasitol (2019) 106:171–208. doi: 10.1016/bs.apar.2019.07.004 31630758 PMC7086480

[141] Muller N von Allmen N. Recent insights into the mucosal reactions associated with giardia lamblia infections. Int J Parasitol (2005) 35(13):1339–47. doi: 10.1016/j.ijpara.2005.07.008 16182298

[142] Troeger H Epple HJ Schneider T Wahnschaffe U Ullrich R Burchard GD. Effect of chronic giardia lamblia infection on epithelial transport and barrier function in human duodenum. Gut. (2007) 56(3):328–35. doi: 10.1136/gut.2006.100198 PMC1856804 16935925

[143] Reynoso-Robles R Ponce-Macotela M Rosas-Lopez LE Ramos-Morales A Martinez-Gordillo MN Gonzalez-Maciel A. The invasive potential of giardia intestinalis in an *in vivo* model. Sci Rep (2015) 5:15168. doi: 10.1038/srep15168 26470844 PMC4607969

[144] Eckmann L Laurent F Langford TD Hetsko ML Smith JR Kagnoff MF. Nitric oxide production by human intestinal epithelial cells and competition for arginine as potential determinants of host defense against the lumen-dwelling pathogen giardia lamblia. J Immunol (2000) 164(3):1478–87. doi: 10.4049/jimmunol.164.3.1478 10640765

[145] Stadelmann B Merino MC Persson L Svard SG. Arginine consumption by the intestinal parasite giardia intestinalis reduces proliferation of intestinal epithelial cells. PloS One (2012) 7(9):e45325. doi: 10.1371/journal.pone.0045325 23028934 PMC3446895

[146] Tako EA Hassimi MF Li E Singer SM. Transcriptomic analysis of the host response to giardia duodenalis infection reveals redundant mechanisms for parasite control. mBio. (2013) 4(6):e00660–13. doi: 10.1128/mBio.00660-13 PMC3892777 24194537

[147] Paerewijck O Maertens B Dreesen L Van Meulder F Peelaers I Ratman D. Interleukin-17 receptor a (IL-17RA) as a central regulator of the protective immune response against giardia. Sci Rep (2017) 7(1):8520. doi: 10.1038/s41598-017-08590-x 28819174 PMC5561107

[148] Manko A Motta JP Cotton JA Feener T Oyeyemi A Vallance BA. Giardia co-infection promotes the secretion of antimicrobial peptides beta-defensin 2 and trefoil factor 3 and attenuates attaching and effacing bacteria-induced intestinal disease. PloS One (2017) 12(6):e0178647. doi: 10.1371/journal.pone.0178647 28622393 PMC5473565

[149] Manko-Prykhoda A Allain T Motta JP Cotton JA Feener T Oyeyemi A. Giardia spp. promote the production of antimicrobial peptides and attenuate disease severity induced by attaching and effacing enteropathogens *via* the induction of the NLRP3 inflammasome. Int J Parasitol (2020) 50(4):263–75. doi: 10.1016/j.ijpara.2019.12.011 32184085

[150] Scott KG Meddings JB Kirk DR Lees-Miller SP Buret AG. Intestinal infection with giardia spp. reduces epithelial barrier function in a myosin light chain kinase-dependent fashion. Gastroenterology. (2002) 123(4):1179–90. doi: 10.1053/gast.2002.36002 12360480

[151] Zhou P Li E Shea-Donohue T Singer SM. Tumour necrosis factor alpha contributes to protection against giardia lamblia infection in mice. Parasite Immunol (2007) 29(7):367–74. doi: 10.1111/j.1365-3024.2007.00953.x PMC2443547 17576366

[152] Chen TL Chen S Wu HW Lee TC Lu YZ Wu LL. Persistent gut barrier damage and commensal bacterial influx following eradication of giardia infection in mice. Gut Pathog (2013) 5(1):26. doi: 10.1186/1757-4749-5-26 23991642 PMC3765889

[153] Bienz M Dai WJ Welle M Gottstein B Muller N. Interleukin-6-deficient mice are highly susceptible to giardia lamblia infection but exhibit normal intestinal immunoglobulin a responses against the parasite. Infect Immun (2003) 71(3):1569–73. doi: 10.1128/IAI.71.3.1569-1573.2003 PMC148820 12595479

[154] Zhou P Li E Zhu N Robertson J Nash T Singer SM. Role of interleukin-6 in the control of acute and chronic giardia lamblia infections in mice. Infect Immun (2003) 71(3):1566–8. doi: 10.1128/IAI.71.3.1566-1568.2003 PMC148826 12595478

[155] Kamda JD Nash TE Singer SM. Giardia duodenalis: dendritic cell defects in IL-6 deficient mice contribute to susceptibility to intestinal infection. Exp Parasitol (2012) 130(3):288–91. doi: 10.1016/j.exppara.2012.01.003 PMC3289762 22248985

[156] Keselman A Li E Maloney J Singer SM. The microbiota contributes to CD8+ T cell activation and nutrient malabsorption following intestinal infection with giardia duodenalis. Infect Immun (2016) 84(10):2853–60. doi: 10.1128/IAI.00348-16 PMC5038064 27456829

[157] Li X Zhang X Gong P Xia F Li L Yang Z. TLR2(-/-) mice display decreased severity of giardiasis *via* enhanced proinflammatory cytokines production dependent on AKT signal pathway. Front Immunol (2017) 8:1186. doi: 10.3389/fimmu.2017.01186 28979269 PMC5611375

[158] Li E Zhou P Petrin Z Singer SM. Mast cell-dependent control of giardia lamblia infections in mice. Infect Immun (2004) 72(11):6642–9. doi: 10.1128/IAI.72.11.6642-6649.2004 PMC523017 15501797

[159] Li E Zhao A Shea-Donohue T Singer SM. Mast cell-mediated changes in smooth muscle contractility during mouse giardiasis. Infect Immun (2007) 75(9):4514–8. doi: 10.1128/IAI.00596-07 PMC1951189 17620354

[160] Li E Tako EA Singer SM. Complement activation by giardia duodenalis parasites through the lectin pathway contributes to mast cell responses and parasite control. Infect Immun (2016) 84(4):1092–9. doi: 10.1128/IAI.00074-16 PMC4807472 26831470

[161] Singer SM Nash TE. T-Cell-dependent control of acute giardia lamblia infections in mice. Infect Immun (2000) 68(1):170–5. doi: 10.1128/IAI.68.1.170-175.2000 PMC97117 10603384

[162] Andersen YS Gillin FD Eckmann L. Adaptive immunity-dependent intestinal hypermotility contributes to host defense against giardia spp. Infect Immun (2006) 74(4):2473–6. doi: 10.1128/IAI.74.4.2473-2476.2006 PMC1418922 16552082

[163] Li E Zhou P Singer SM. Neuronal nitric oxide synthase is necessary for elimination of giardia lamblia infections in mice. J Immunol (2006) 176(1):516–21. doi: 10.4049/jimmunol.176.1.516 PMC2585514 16365445

[164] Scott KG Yu LC Buret AG. Role of CD8+ and CD4+ T lymphocytes in jejunal mucosal injury during murine giardiasis. Infect Immun (2004) 72(6):3536–42. doi: 10.1128/IAI.72.6.3536-3542.2004 PMC415705 15155662

[165] Bouzid M Hunter PR McDonald V Elwin K Chalmers RM Tyler KM. A new heterogeneous family of telomerically encoded cryptosporidium proteins. Evol Appl (2013) 6(2):207–17. doi: 10.1111/j.1752-4571.2012.00277.x PMC3586618 23467513

[166] Huang DB White AC. An updated review on cryptosporidium and giardia. Gastroenterol Clin North Am (2006) 35(2):291–314. doi: 10.1016/j.gtc.2006.03.006 16880067

[167] Farthing MJ. Clinical aspects of human cryptosporidiosis. Contrib Microbiol (2000) 6:50–74. doi: 10.1159/000060368 10943507

[168] Collaborators GBDDD. Estimates of global, regional, and national morbidity, mortality, and aetiologies of diarrhoeal diseases: a systematic analysis for the global burden of disease study 2015. Lancet Infect Dis (2017) 17(9):909–48. doi: 10.1016/S1473-3099(17)30276-1 PMC5589208 28579426

[169] Crawford CK Kol A. The mucosal innate immune response to cryptosporidium parvum, a global one health issue. Front Cell Infect Microbiol (2021) 11:689401. doi: 10.3389/fcimb.2021.689401 34113580 PMC8185216

[170] McDonald V Korbel DS Barakat FM Choudhry N Petry F. Innate immune responses against cryptosporidium parvum infection. Parasite Immunol (2013) 35(2):55–64. doi: 10.1111/pim.12020 23173616

[171] Laurent F Lacroix-Lamande S. Innate immune responses play a key role in controlling infection of the intestinal epithelium by cryptosporidium. Int J Parasitol (2017) 47(12):711–21. doi: 10.1016/j.ijpara.2017.08.001 28893638

[172] Lantier L Lacroix-Lamande S Potiron L Metton C Drouet F Guesdon W. Intestinal CD103+ dendritic cells are key players in the innate immune control of cryptosporidium parvum infection in neonatal mice. PloS Pathog (2013) 9(12):e1003801. doi: 10.1371/journal.ppat.1003801 24367259 PMC3868524

[173] Lacroix-Lamande S Mancassola R Naciri M Laurent F. Role of gamma interferon in chemokine expression in the ileum of mice and in a murine intestinal epithelial cell line after cryptosporidium parvum infection. Infect Immun (2002) 70(4):2090–9. doi: 10.1128/IAI.70.4.2090-2099.2002 PMC127832 11895975

[174] Pantenburg B Dann SM Wang HC Robinson P Castellanos-Gonzalez A Lewis DE. Intestinal immune response to human cryptosporidium sp. infection. Infect Immun (2008) 76(1):23–9. doi: 10.1128/IAI.00960-07 PMC2223661 17967863

[175] Chen XM Levine SA Splinter PL Tietz PS Ganong AL Jobin C. Cryptosporidium parvum activates nuclear factor kappaB in biliary epithelia preventing epithelial cell apoptosis. Gastroenterology. (2001) 120(7):1774–83. doi: 10.1053/gast.2001.24850 11375958

[176] Chen XM O'Hara SP Nelson JB Splinter PL Small AJ Tietz PS. Multiple TLRs are expressed in human cholangiocytes and mediate host epithelial defense responses to cryptosporidium parvum *via* activation of NF-kappaB. J Immunol (2005) 175(11):7447–56. doi: 10.4049/jimmunol.175.11.7447 16301652

[177] Choudhry N Petry F van Rooijen N McDonald V. A protective role for interleukin 18 in interferon gamma-mediated innate immunity to cryptosporidium parvum that is independent of natural killer cells. J Infect Dis (2012) 206(1):117–24. doi: 10.1093/infdis/jis300 22517912

[178] Korbel DS Barakat FM Di Santo JP McDonald V. CD4+ T cells are not essential for control of early acute cryptosporidium parvum infection in neonatal mice. Infect Immun (2011) 79(4):1647–53. doi: 10.1128/IAI.00922-10 PMC3067537 21282414

[179] Urban JF Jr. Fayer R Chen SJ Gause WC Gately MK Finkelman FD. IL-12 protects immunocompetent and immunodeficient neonatal mice against infection with cryptosporidium parvum. J Immunol (1996) 156(1):263–8. 8598471

[180] Mead JR You X. Susceptibility differences to cryptosporidium parvum infection in two strains of gamma interferon knockout mice. J Parasitol (1998) 84(5):1045–8. doi: 10.2307/3284643 9794653

[181] Ehigiator HN McNair N Mead JR. Cryptosporidium parvum: the contribution of Th1-inducing pathways to the resolution of infection in mice. Exp Parasitol (2007) 115(2):107–13. doi: 10.1016/j.exppara.2006.07.001 16920103

[182] Tessema TS Schwamb B Lochner M Forster I Jakobi V Petry F. Dynamics of gut mucosal and systemic Th1/Th2 cytokine responses in interferon-gamma and interleukin-12p40 knock out mice during primary and challenge cryptosporidium parvum infection. Immunobiology. (2009) 214(6):454–66. doi: 10.1016/j.imbio.2008.11.015 19155092

[183] Sateriale A Gullicksrud JA Engiles JB McLeod BI Kugler EM Henao-Mejia J. The intestinal parasite Cryptosporidium is controlled by an enterocyte intrinsic inflammasome that depends on NLRP6. Proc Natl Acad Sci USA (2021) 118(2):e2007807118. doi: 10.1073/pnas.2007807118 33372132 PMC7812745

[184] Gullicksrud JA Sateriale A Engiles JB Gibson AR Shaw S Hutchins ZA. Enterocyte-innate lymphoid cell crosstalk drives early IFN-gamma-mediated control of cryptosporidium. Mucosal Immunol (2022) 15(2):362–72. doi: 10.1038/s41385-021-00468-6 PMC8881313 34750455

[185] Ungar BL Kao TC Burris JA Finkelman FD. Cryptosporidium infection in an adult mouse model. independent roles for IFN-gamma and CD4+ T lymphocytes in protective immunity. J Immunol (1991) 147(3):1014–22. 1677668

[186] McDonald V Bancroft GJ. Mechanisms of innate and acquired resistance to cryptosporidium parvum infection in SCID mice. Parasite Immunol (1994) 16(6):315–20. doi: 10.1111/j.1365-3024.1994.tb00354.x 7970868

[187] Hayward AR Chmura K Cosyns M. Interferon-gamma is required for innate immunity to cryptosporidium parvum in mice. J Infect Dis (2000) 182(3):1001–4. doi: 10.1086/315802 10950807

[188] Robinson P Okhuysen PC Chappell CL Lewis DE Shahab I Lahoti S. Expression of IL-15 and IL-4 in IFN-gamma-independent control of experimental human cryptosporidium parvum infection. Cytokine. (2001) 15(1):39–46. doi: 10.1006/cyto.2001.0888 11509007

[189] Barakat FM McDonald V Di Santo JP Korbel DS. Roles for NK cells and an NK cell-independent source of intestinal gamma interferon for innate immunity to cryptosporidium parvum infection. Infect Immun (2009) 77(11):5044–9. doi: 10.1128/IAI.00377-09 PMC2772539 19687195

[190] McDonald SA O'Grady JE Bajaj-Elliott M Notley CA Alexander J Brombacher F. Protection against the early acute phase of cryptosporidium parvum infection conferred by interleukin-4-induced expression of T helper 1 cytokines. J Infect Dis (2004) 190(5):1019–25. doi: 10.1086/422761 15295711

[191] Enriquez FJ Sterling CR. Role of CD4+ TH1- and TH2-cell-secreted cytokines in cryptosporidiosis. Folia Parasitol (Praha). (1993) 40(4):307–11. 7912219

[192] Aguirre SA Mason PH Perryman LE. Susceptibility of major histocompatibility complex (MHC) class I- and MHC class II-deficient mice to cryptosporidium parvum infection. Infect Immun (1994) 62(2):697–9. doi: 10.1128/iai.62.2.697-699.1994 PMC186160 7905464

[193] Chen W Harp JA Harmsen AG. Cryptosporidium parvum infection in gene-targeted b cell-deficient mice. J Parasitol (2003) 89(2):391–3. doi: 10.1645/0022-3395(2003)089[0391:CPIIGB]2.0.CO;2 12760662

[194] Frost FJ Roberts M Kunde TR Craun G Tollestrup K Harter L. How clean must our drinking water be: the importance of protective immunity. J Infect Dis (2005) 191(5):809–14. doi: 10.1086/427561 15688300

[195] Frost FJ Tollestrup K Craun GF Fairley CK Sinclair MI Kunde TR. Protective immunity associated with a strong serological response to a cryptosporidium-specific antigen group, in HIV-infected individuals. J Infect Dis (2005) 192(4):618–21. doi: 10.1086/431681 16028130

[196] Dumaine JE Sateriale A Gibson AR Reddy AG Gullicksrud JA Hunter EN. The enteric pathogen cryptosporidium parvum exports proteins into the cytosol of the infected host cell. Elife (2021) 10 :e70451. doi: 10.7554/eLife.70451 34866573 PMC8687662

[197] Wei Y Gao J Kou Y Meng L Zheng X Liang M. Commensal bacteria impact a protozoan's integration into the murine gut microbiota in a dietary nutrient-dependent manner. Appl Environ Microbiol (2020) 86(11):e00303-20. doi: 10.1128/AEM.00303-20 32198171 PMC7237772

[198] Audebert C Even G Cian A Blastocystis Investigation G Loywick A Merlin S. Colonization with the enteric protozoa blastocystis is associated with increased diversity of human gut bacterial microbiota. Sci Rep (2016) 6:25255. doi: 10.1038/srep25255 27147260 PMC4857090

[199] Hatter JA Kouche YM Melchor SJ Ng K Bouley DM Boothroyd JC. Toxoplasma gondii infection triggers chronic cachexia and sustained commensal dysbiosis in mice. PloS One (2018) 13(10):e0204895. doi: 10.1371/journal.pone.0204895 30379866 PMC6209157

[200] Iebba V Santangelo F Totino V Pantanella F Monsia A Di Cristanziano V. Gut microbiota related to giardia duodenalis, entamoeba spp. and blastocystis hominis infections in humans from cote d'Ivoire. J Infect Dev Ctries. (2016) 10(9):1035–41. doi: 10.3855/jidc.8179 27694739

[201] Barash NR Maloney JG Singer SM Dawson SC. Giardia alters commensal microbial diversity throughout the murine gut. Infect Immun (2017) 85(6):e00948. doi: 10.1128/IAI.00948-16 28396324 PMC5442636

[202] Beatty JK Akierman SV Motta JP Muise S Workentine ML Harrison JJ. Giardia duodenalis induces pathogenic dysbiosis of human intestinal microbiota biofilms. Int J Parasitol (2017) 47(6):311–26. doi: 10.1016/j.ijpara.2016.11.010 28237889

[203] McKenney EA Greene LK Drea CM Yoder AD. Down for the count: Cryptosporidium infection depletes the gut microbiome in coquerel's sifakas. Microb Ecol Health Dis (2017) 28(1):1335165. doi: 10.1080/16512235.2017.1335165 28740461 PMC5508644

[204] Mammeri M Chevillot A Thomas M Julien C Auclair E Pollet T. Cryptosporidium parvum-infected neonatal mice show gut microbiota remodelling using high-throughput sequencing analysis: Preliminary results. Acta Parasitol (2019) 64(2):268–75. doi: 10.2478/s11686-019-00044-w 30915719

[205] Hand TW Dos Santos LM Bouladoux N Molloy MJ Pagan AJ Pepper M. Acute gastrointestinal infection induces long-lived microbiota-specific T cell responses. Science. (2012) 337(6101):1553–6. doi: 10.1126/science.1220961 PMC3784339 22923434

[206] Melgar S Karlsson A Michaelsson E. Acute colitis induced by dextran sulfate sodium progresses to chronicity in C57BL/6 but not in BALB/c mice: correlation between symptoms and inflammation. Am J Physiol Gastrointest Liver Physiol (2005) 288(6):G1328–38. doi: 10.1152/ajpgi.00467.2004 15637179

[207] Huang J DeGraves FJ Lenz SD Gao D Feng P Li D. The quantity of nitric oxide released by macrophages regulates chlamydia-induced disease. Proc Natl Acad Sci U S A. (2002) 99(6):3914–9. doi: 10.1073/pnas.062578399 PMC122623 11904441

[208] Mills CD Kincaid K Alt JM Heilman MJ Hill AM. M-1/M-2 macrophages and the Th1/Th2 paradigm. J Immunol (2000) 164(12):6166–73. doi: 10.4049/jimmunol.164.12.6166 10843666

[209] Falkow S. Is persistent bacterial infection good for your health? Cell (2006) 124(4):699–702. doi: 10.1016/j.cell.2006.02.004 16497581

[210] Smith PM Howitt MR Panikov N Michaud M Gallini CA Bohlooly YM. The microbial metabolites, short-chain fatty acids, regulate colonic treg cell homeostasis. Science. (2013) 341(6145):569–73. doi: 10.1126/science.1241165 PMC3807819 23828891

[211] Kamada N Nunez G. Role of the gut microbiota in the development and function of lymphoid cells. J Immunol (2013) 190(4):1389–95. doi: 10.4049/jimmunol.1203100 PMC3564600 23378581

[212] Pandiyan P Bhaskaran N Zou M Schneider E Jayaraman S Huehn J. Microbiome dependent regulation of tregs and Th17 cells in mucosa. Front Immunol (2019) 10:426. doi: 10.3389/fimmu.2019.00426 30906299 PMC6419713

[213] Al Bander Z Nitert MD Mousa A Naderpoor N. The gut microbiota and inflammation: An overview. Int J Environ Res Public Health (2020) 17(20):7618. doi: 10.3390/ijerph17207618 PMC7589951 33086688

[214] Sokurenko EV Hasty DL Dykhuizen DE. Pathoadaptive mutations: gene loss and variation in bacterial pathogens. Trends Microbiol (1999) 7(5):191–5. doi: 10.1016/S0966-842X(99)01493-6 10354593

[215] Gal-Mor O Finlay BB. Pathogenicity islands: a molecular toolbox for bacterial virulence. Cell Microbiol (2006) 8(11):1707–19. doi: 10.1111/j.1462-5822.2006.00794.x 16939533

[216] Tenaillon O Skurnik D Picard B Denamur E. The population genetics of commensal escherichia coli. Nat Rev Microbiol (2010) 8(3):207–17. doi: 10.1038/nrmicro2298 20157339

[217] Crossman LC Chaudhuri RR Beatson SA Wells TJ Desvaux M Cunningham AF. A commensal gone bad: complete genome sequence of the prototypical enterotoxigenic escherichia coli strain H10407. J Bacteriol (2010) 192(21):5822–31. doi: 10.1128/JB.00710-10 PMC2953697 20802035

[218] Kohler CD Dobrindt U. What defines extraintestinal pathogenic escherichia coli? Int J Med Microbiol (2011) 301(8):642–7. doi: 10.1016/j.ijmm.2011.09.006 21982038

[219] Proenca JT Barral DC Gordo I. Commensal-to-pathogen transition: One-single transposon insertion results in two pathoadaptive traits in escherichia coli -macrophage interaction. Sci Rep (2017) 7(1):4504. doi: 10.1038/s41598-017-04081-1 28674418 PMC5495878

[220] Ehrlich GD Hiller NL Hu FZ. What makes pathogens pathogenic. Genome Biol (2008) 9(6):225. doi: 10.1186/gb-2008-9-6-225 18598378 PMC2481411

[221] Duraisingh MT Horn D. Epigenetic regulation of virulence gene expression in parasitic Protozoa. Cell Host Microbe (2016) 19(5):629–40. doi: 10.1016/j.chom.2016.04.020 PMC5061559 27173931

[222] Goodacre R. Metabolomics of a superorganism. J Nutr (2007) 137(1 Suppl):259S–66S. doi: 10.1093/jn/137.1.259S 17182837

